# An analysis on the role of glucagon-like peptide 1 receptor agonists in cognitive and mental health disorders

**DOI:** 10.1038/s44220-025-00390-x

**Published:** 2025-02-13

**Authors:** Riccardo De Giorgi, Ana Ghenciulescu, Oliwia Dziwisz, Maxime Taquet, Amanda I Adler, Ivan Koychev, Rachel Upthegrove, Marco Solmi, Robert McCutcheon, Toby Pillinger, Philip J Cowen, Catherine J Harmer

**Affiliations:** 1 Department of Psychiatry, University of Oxford, Warneford Hospital, Warneford Lane, Oxford, OX3 7JX, United Kingdom; 2 Oxford Health NHS Foundation Trust, Warneford Hospital, Warneford Lane, Oxford, OX3 7JX, United Kingdom; 3 Diabetes Trials Unit, Radcliffe Department of Medicine, University of Oxford, John Radcliffe Hospital, Headley Way, Oxford, OX3 9DU, United Kingdom; 4 Oxford University Hospital NHS Foundation Trust, John Radcliffe Hospital, Headley Way, Oxford, OX3 9DU, United Kingdom; 5 Institute for Mental Health, University of Birmingham, Birmingham UK; Birmingham Early Intervention Service, Birmingham Womens and Childrens NHS Foundation Trust; 6 SCIENCES lab, Department of Psychiatry, University of Ottawa, Ontario, Canada; 7 Regional Centre for the Treatment of Eating Disorders and On Track: The Champlain First Episode Psychosis Program, Department of Mental Health, The Ottawa Hospital, Ontario, Canada; 8 Ottawa Hospital Research Institute (OHRI) Clinical Epidemiology Program University of Ottawa, Ottawa Ontario; 9 Department of Child and Adolescent Psychiatry, Charité Universitätsmedizin, Berlin, Germany; 10 Department of Psychosis Studies, Institute of Psychiatry, Psychology and Neuroscience, London, UK, SE5 8AF

## Abstract

Glucagon-like peptide-1 receptor agonists (GLP-1RAs) are novel drugs approved for diabetes and obesity. They are acknowledged as a major scientific breakthrough. In addition to their metabolic effects, these medications act on other bodily systems involved in the physiopathology of various neurological and psychiatric disorders. Several stakeholders are calling for more research to investigate the repurposing potential of GLP-1RAs in cognitive and mental disorders, while others advocate for a better assessment of their safety profile from a neuropsychiatric perspective. In this review, we searched for relevant literature on the effects of GLP-1RAs across a range of illnesses, gathering and describing the available pre-clinical/mechanistic (278 studies) and clinical (96 studies) evidence for cognitive disorders, substance use disorders, psychotic disorders, mood and anxiety disorders, eating disorders, and others. By leveraging translational insights from these data, we consider potential implications for clinical practice and propose avenues for further research.

## Introduction

Glucagon-like peptide-1 receptor agonists (GLP-1RAs, also known as “incretin mimetics”) are a class of medications licensed for the treatment of type 2 diabetes mellitus (T2DM) and obesity^1^. These drugs fall within two categories: human GLP-1 backbone agents (i.e., albiglutide, dulaglutide, liraglutide, and semaglutide) and exendin-4 backbone agents (i.e., exenatide, lixisenatide, and tirzepatide – the latter activating both GLP-1 and glucose-dependent insulinotropic polypeptide GIP) receptors)^1^. GLP-1 and GIP are incretin hormones that stimulate insulin secretion after an oral glucose load by binding GLP-1R, but both are rapidly inactivated by the enzyme dipeptidyl peptidase-4 (DPP-4). GLP-1RAs activate GLP-1R similarly to GLP-1, but they are resistant to the activity of DPP-4. Ultimately, GLP-1RAs enhance insulin excretion, leading to the inhibition of glucagon production by pancreatic α-cells when blood sugar levels are high as well as a decrease of pancreatic β-cell apoptosis and an increase in their proliferation. Further, these drugs delay gastric emptying and appear to increase satiety due to direct activity on the hypothalamus and brain stem. Numerous studies have investigated the expression patterns of endogenous GLP-1 and GLP-1R in the central and peripheral nervous systems (CNS, PNS), with a consensus that these are expressed on neurons and found in most areas of the brain and gut-brain axis^2^. Specifically, beyond the enteroendocrine L-cells of the intestine, GLP-1 is also produced as a neuropeptide by the pre-proglucagon (PPG) neurons in the brain stem^3^. Although some GLP-1RAs do not seem to naturally cross the blood-brain barrier, they may still reach relevant brain areas via circumventricular sites and, possibly, via active transporters^4,5^. The implications of centrally-produced, neuromodulatory GLP-1 in the context of GLP-RAs are uncertain, since the degree to which signals from PPG neurons/endogenous GLP-1 system and GLP-1RAs activity converge on shared downstream targets is unclear^6^ and may in fact occur independently^7^. Most GLP-1RAs, aside from a new oral formulation of semaglutide (Rybelsus® tablets), are administered subcutaneously via pen-like devices (once-daily to once-weekly) due to poor oral bioavailability, and all are renally excreted^1^. Nausea, vomiting, dyspepsia and diarrhoea are common side effects; uncommon or unconfirmed more severe reactions may include acute kidney injury, hypoglycaemia, thyroid neoplasia, and acute pancreatitis.

Because of their substantial benefit on some of the most highly prevalent disorders worldwide, GLP-1RA have been hailed as “game changers”^8,9^ and “breakthrough drugs”^10^, with an estimated market value of USD 22.4 billion in 2022 and a compound annual growth rate of around 9.6% between 2023-2032^11^. They are being extensively used (i.e., prescribed both in-label and off-label) and misused (i.e., obtained without prescription online) for weight loss in the general population, under the limelight of a so-called “media frenzy”^12^. Such widespread usage has led to a severe and prolonged international shortage of these drugs^13,14^, with consequent lack of access to treatment for diabetic patients^15^ and the urgent need to issue guidelines for alternative treatments^16^.

Several major randomised controlled trials (RCTs) have confirmed the efficacy and safety of GLP-1RAs in adults with diabetes^17^ and obesity^18^, and more recently in child and adolescent populations living with obesity^19,20^. Importantly, these medications lead to a considerable reduction of cardiovascular morbidity^21^ and population-level all-cause mortality^22^. Other trials are investigating their metabolic and non-metabolic (i.e., disease-specific) effects in a variety of chronic illnesses including kidney and liver disorders, Alzheimer’s dementia, and schizophrenia^23,24^. Based on several putative modes of action under investigation (e.g., neuroprotective and anti-inflammatory properties, regulation of reward pathways) there is an emerging consensus that GLP-1RAs could be repurposed for use in neuropsychiatric conditions^25–32^. In this comprehensive overview (see Methods and search methodology in Supplementary Information S1), we aim to identify and describe pre-clinical, mechanistic, and clinical studies on the effects of GLP-1RAs in cognitive and mental health disorders, and to provide a summary of available evidence and future perspectives. Evidence was reported according to the neuropsychiatric condition under investigation: cognitive disorders (dementia, Parkinson’s disease), substance use disorders, psychotic disorders, mood and anxiety disorders, and eating disorders – each subdivided into pre-clinical/mechanistic evidence and clinical evidence, the latter reported following hierarchy of evidence (i.e., meta-analyses, clinical trials, observational studies, case series). Miscellaneous studies (e.g., reporting on any psychiatric adverse outcomes) as well as ongoing/planned trials were reported in Supplementary Information S2 and Supplementary Information S3 respectively.

## Results

**Extended Data Figure 1:**
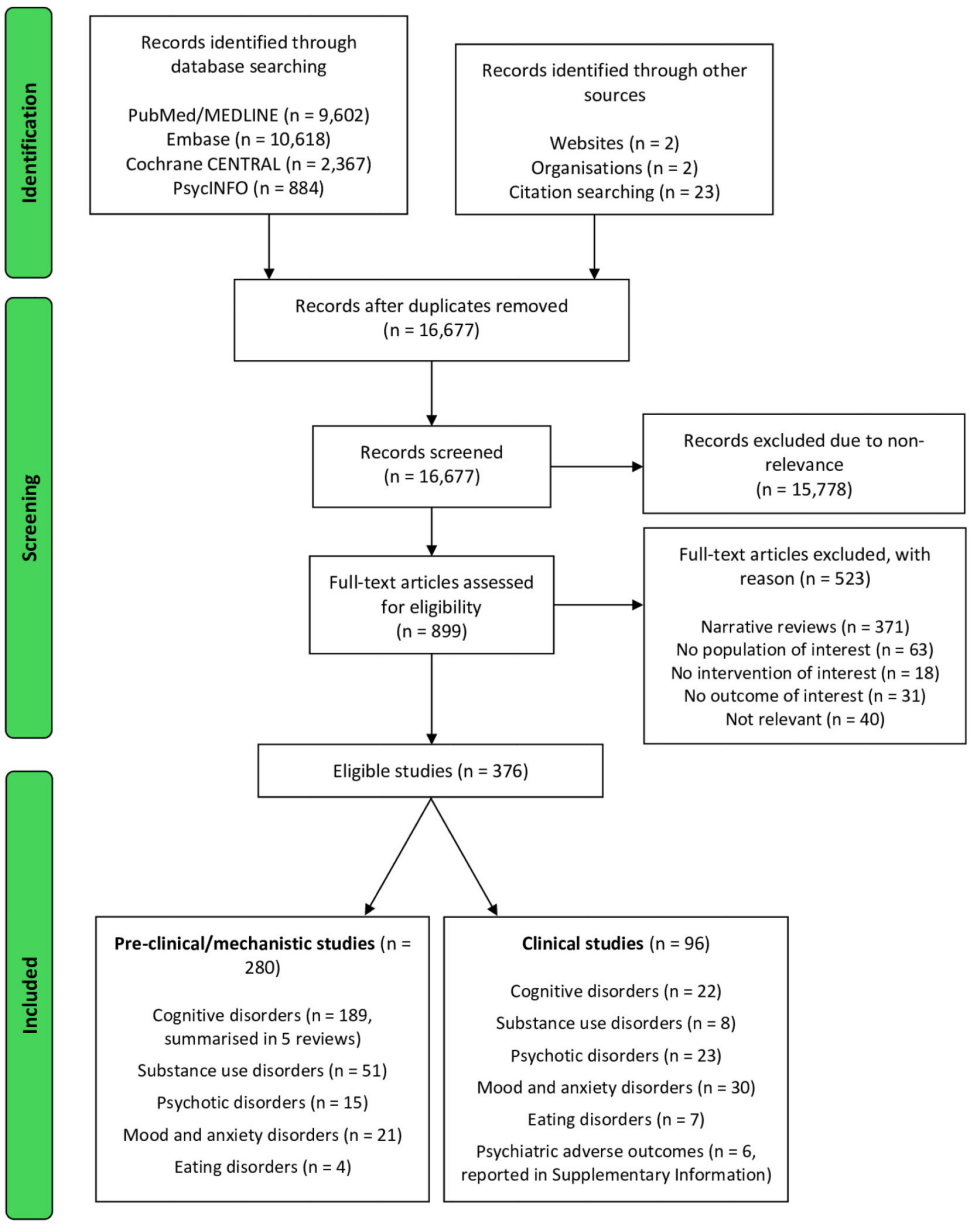
Flow chart of the review process.

The initial search yielded 23,496 records of which 6,821 were duplicates. Screening of 16,675 titles and abstracts led to the removal of 15,778 non-relevant studies. A further 523 articles were excluded on eligibility assessment of 897 full texts. Eventually, 374 studies were eligible for inclusion in the review (Extended Data Figure 1).

### Cognitive Disorders

#### Pre-clinical/mechanistic studies

Our search retrieved a high number (N = 189) of pre-clinical or mechanistic studies assessing the possible effects of GLP-1RAs on cognitive disorders, which cannot be described in the main text of this article due to space constraints (see Supplementary Information S4). Here, we therefore only report the 5 more recent and inclusive reviews that summarise such evidence. A meta-analysis of 26 animal studies showed that GLP-1RAs improved learning and memory in rodent models of Alzheimer’s disease (AD), possibly by decreasing brain levels of Aβ-amyloid deposition and phosphorylated tau^33^. There is also evidence for mechanisms involving a reduction of neuroinflammation, an increase in synaptic functioning, as well as the restoration of brain pathways of insulin signalling that may lead to improved memory formation and therefore a positive effect in AD and Parkinson’s disease (PD)^34^. Brain insulin resistance may indeed play a role in the pathophysiology of cognitive disorders, and addressing this may be a mechanism via which GLP-1RAs act pro-cognitively^35^. GLP-1R activation of neuroprotective pathways in neurons, microglia, and astrocytes has also been reported: improvements in overall cognition, learning, and motor function potentially associated with GLP-1RA administration in AD and PD may be mediated not only by their amyloid pathology-ameliorating properties (Aβ, tau, and α-synuclein), but also the suppression of Ca^2+^ deregulation and endoplasmic reticulum stress, anti-inflammatory activity, blockage of oxidative stress, mitochondrial dysfunction and apoptosis pathways, enhancements in the neuronal insulin sensitivity and energy metabolism, functional improvements in autophagy and mitophagy, elevated BDNF and glial cell line-derived neurotrophic factor (GDNF) synthesis, as well as neurogenesis^36^. Other neuroprotective mechanisms potentially involved in the treatment of cognitive disorders as well as cerebrovascular disease and epilepsy suggest that GLP-1RAs can enhance the viability of neurons and restore neurite outgrowth by stimulating neurotrophic factors, thus increasing subventricular zone progenitor cells, decreasing apoptosis and the level of pro-inflammatory factors, and strengthening the blood-brain barrier^37^.

#### Clinical studies

A total of 22 completed clinical studies were identified (Table 1, Extended Data Tables 1-2), with another 8 clinical trials still ongoing (Supplementary Information S3).

Four meta-analyses pooled both randomised and non-randomised evidence to assess the effects of GLP-1RAs on dementia risk or cognitive outcomes from studies conducted in people with a background diagnosis of T2DM or obesity^38–41^. A meta-analysis of 3 RCTs^42–44^ and 2mor prospective cohort studies^45,46^ comprising 7,732 adults with T2DM did not observe any effect on cognition, as measured via mini-mental state examination (MMSE) or Montreal cognitive assessment (MoCA), of GLP-1RAs over several months compared to baseline^38^. A pooled analysis of 3 longer term RCTs^22,47,48^ following 15,820 T2DM patients up to 3.8 years showed a reduced risk of dementia for semaglutide and liraglutide compared to placebo^39^. The same paper also included a nested case-control component of 120,054 patients with T2DM followed for 7.4 years and observed a lower association between dementia and exposure to GLP-1RAs compared to other antidiabetics^39^. This finding was further supported when pooled with further observational data^49,50^ to a total of 210,521 people with T2DM up to 7.4 years on any GLP-1RA^40^. Finally, a recent network meta-analysis that compared cognitive outcomes with various antidiabetic agents in patients with type 2 diabetes observed that GLP-1RAs ranked second after sodium-glucose cotransporter-2 inhibitors (SGLT-2I) for reducing dementia risk. However, this meta-analysis only included 1 RCT^43^ and 1 case-control study (Akimoto 2020) for GLP-1RAs (but not the more recent semaglutide)^41^.

All clinical trials for dementia outcomes identified by our search^42–44^ had been included in the meta-analyses above. Among these trials, one involving 36 patients with T2DM did not show any difference from baseline on the MMSE and MoCA after liraglutide at 16 weeks – though all participants had preserved cognitive function at baseline, while an improvement on tests for delayed memory (possibly mediated by left hippocampal activation), attention, and executive function was noted^42^.

However, we further retrieved 4 RCTs in Parkinson’s disease looking at both motor and non-motor outcomes, but results were inconsistent depending on the different scales used within the same trial; overall, 1 showed a more beneficial profile^51^, while the other 3 did not find any consistently positive effect^52–54^.

Among the observational studies^39,45,46,49,50,55^ comprised by the meta-analyses, two are worth a separate mention. One observed a positive association between liraglutide use at 12 weeks and improved MMSE in 47 adults with T2DM, which correlated with increased task (verbal fluency)-based activation of the dorsolateral prefrontal and orbitofrontal cortex, while several other cognitive tests were not affected^45^. In another study, the same treatment in 19 obese subjects with diabetes was associated with improved MoCA score, olfactory test total score, and enhanced odour-induced right parahippocampus activation^46^. Moreover, we retrieved other relevant records^56–58^: two large (N = 133,318 and N = 342,608 respectively) cohort studies in people with diabetes across 6-13 years noted a beneficial association between GLP-1RAs prescriptions compared to non-prescription and lower diagnoses of dementia^57,58^, whilst a small (N = 154 patients with T2DM) and shorter (~12months) cross-sectional investigation of GLP-1RAs in addition to metformin, compared to metformin alone, observed better MoCA scores in the former group^56^.

No studies investigating possible interactions between GLP-1RAs and antidementia drugs were found.

In summary, there is a considerable number of clinical studies reporting the potential benefit of GLP-1RAs for use in cognitive disorders, including dementias and Parkinson’s disease, though the majority are observational and can only suggest association. Such evidence, however, is supported by many relevant pre-clinical/mechanistic studies highlighting the neuroprotective and anti-inflammatory activity of these medications. Conversely, we found little evidence that GLP-1RAs may cause or exacerbate cognitive impairment, which is of importance to patients who may need taking these medications for their currently licensed (and expanding) indications.

### Substance Use Disorders

#### Pre-clinical/mechanistic studies

A large body of pre-clinical and mechanistic literature is available regarding the putative effects of GLP-1RAs on substance misuse (Supplementary Information S5): 24 for alcohol^59–82^, 8 for opiates^63,83–89^, 16 for stimulants including cocaine and amphetamines^90–105^, and 4 for nicotine^106–109^. A large proportion of these studies reported on the impact of GLP-1RAs on dopaminergic neurotransmission responsible for reward processing – which could contribute to their efficacy as anti-obesity medications by means of a reduction of food-related incentive^110^.

##### Alcohol

Several studies investigating exendin-4, liraglutide, dulaglutide, and semaglutide in rats/mice found a decrease in alcohol use, which was mediated by mesolimbic dopamine pathways involving the nucleus accumbens (NAc), the ventral tegmental area (VTA) and ventral hippocampus, the dorso-lateral septum (DLS), and the nucleus of the solitary tract (NST)^59–61,63–69,72,74–77,79–82,106^. One study replicated such positive findings in non-human primates^78^. It has also been suggested that GLP-1RAs may affect alcohol misuse and withdrawal symptoms by modulating anxiogenic mechanisms in rats^73^. Another study showed no synergistic activity of the antismoking agents, bupropion and varenicline, when administered to rats in addition to semaglutide to reduce alcohol intake^62^. Finally, a *post-mortem* analysis of human brain samples showed increased hippocampal expression of genes encoding for GLP-1R in individuals with severe alcohol use disorder compared to controls^71^.

##### Opiate

Exendin-4 and liraglutide reduced cue- and drug-induced opiate seeking behaviour in rat/mice across several studies^83–88^. Only one study did not identify any benefit of GLP-1RAs in animal models of opiate misuse, though this same study had shown a positive effect for alcohol misuse^63^. An investigation of the dual GLP-1R and neuropeptide Y2-receptor agonist, GEP44 found that this drug attenuated opioid-taking and -seeking at a dose that did not suppress food intake in rats^89^.

##### Cocaine and amphetamines (stimulants)

All animal studies retrieved for stimulants misuse involved exendin-4 among GLP-1RAs and highlighted a reduction of cocaine and amphetamines intake and end-effects (e.g., increased locomotor activity) via modulation of dopaminergic transmission in areas including the NAc and the VTA^91–94,96–103,105^, as well as modulation of inflammatory mechanisms^104^. One genetic study described an enhanced effect on cocaine use in GLP-1R knock-out mice achieved via viral-vector delivery of the gene encoding for GLP-1R to the DLS^95^. In humans, intravenous cocaine injection was shown to decrease plasma GLP-1 concentration, while endogenous GLP-1 was associated with subjective responses to cocaine^90^.

##### Nicotine

Only two pre-clinical investigations on the effects of GLP-1RAs in nicotine misuse were retrieved, both showing less nicotine use and related outcomes (e.g., withdrawal-induced hyperphagia) for liraglutide^108^ and exendin-4 possibly related to dopamine regulation^106^. Moreover, liraglutide appears to diminish nicotine-induced dopamine signalling in the nucleus accumbens^107^. An optogenetic stimulation of GLP-1Rs in habenular circuits was also shown to abolish nicotine reward and decrease nicotine intake in mice^109^.

#### Clinical studies

Compared to the considerable amount of pre-clinical and mechanistic research reported above, we identified few clinical studies of GLP-1RAs for substance use disorders (Table 2): 3 for alcohol^111–113^, 1 for cannabis^114^, 2 for cocaine^115,116^, and 2 for nicotine^117,118^, while no article about opiates or amphetamines was retrieved. However, we found another 9 clinical trials that are ongoing: 6 for alcohol, 1 for opiates, and 2 for nicotine (Supplementary Information S3).

##### Alcohol

A recent 26-week RCT of 127 people with alcohol use disorders found a positive effect of exenatide compared to placebo in obese people only^111^. A similar beneficial association was seen in an observational study of semaglutide in 83,825 patients with obesity and 598,803 patients with T2DM over 12 months^112^, as well as in 87,676 new users of GLP-1RAs or DPP-4 inhibitors over 4 years^113^.

##### Cannabis

While no pre-clinical or mechanistic study has considered GLP-1RAs for cannabis misuse so far, a large epidemiological investigation has recently noted an association between semaglutide use and fewer cannabis use disorders in both patients with T2DM (N = 596,045) and obesity (N = 85,223) over a 1-year follow-up^114^.

##### Cocaine

Only limited clinical evidence is available for GLP-1RAs in cocaine misuse: a small (N = 13) proof-of-concept trial across 2 days showed that exenatide compared to placebo did not reduce the number of self-administered cocaine infusions^115^, while a case series of three individuals with cocaine use disorder highlighted the feasibility and safety of using the same drug over 6 weeks, though no efficacy measures were reported^116^.

##### Nicotine

A trial of 84 prediabetic and overweight smokers found that exenatide was superior to placebo in terms of nicotine abstinence rates at 6 weeks^118^. However, a more recent RCT of 255 adults with nicotine dependence did not show any effect of adjunctive dulaglutide compared to standard of care (i.e., behavioural counselling with varenicline) on cigarette abstinence over 12 weeks of treatment^117^.

No studies investigating possible interactions between GLP-1RAs and anti-addiction drugs were found.

Overall, compared to the large and growing amount of pre-clinical/mechanistic evidence highlighting the reward-modulating and thus potentially anti-addictive properties of GLP-1RAs, only few studies have investigated thus far the potential use of these medications in clinical populations with alcohol or other substance use disorders. Because this is an area with significant unmet needs, especially in terms of pharmacological treatment options, further research investment is warranted.

### Psychotic Disorders

#### Pre-clinical/mechanistic studies

Several pre-clinical and mechanistic studies examined the possible effects of GLP-1RAs in psychotic disorders (Supplementary Information S5). In animal models of psychosis, liraglutide administration consistently led to a reduction of psychotic-like behaviour^119–121^, which was also associated with increased BDNF, CREB/p-CREB, and Trk-B expression in the hippocampus and prefrontal cortex^120^, and reduced serum and hippocampal TNF and oxidative stress^121^.

Several animal studies investigated the effects of liraglutide^122–127^ and exendin-4^126,128^ on metabolic side effects (e.g., hyperglycaemia, hyperlipidaemia, weight gain) of atypical antipsychotics including olanzapine, quetiapine, brexpiprazole, and clozapine. All^122,124–128^ but one^123^ study showed a benefit on metabolic parameters. Two studies also displayed concomitant improvements in cognitive measures of recognition and working memory^122^ and depressive-like behaviour in rats administered antipsychotics^127^. A similarly positive effect on glucose metabolism was observed in mice exposed to clozapine and the non-peptidic GLP-1RA Boc5^129^.

Three studies explored mechanistic associations between GLP-1 functioning, psychosis, and antipsychotic treatment in humans. Low levels of serum GLP-1 were reported in 260 patients with a diagnosis of first-episode psychosis compared to healthy controls^130^. Serum GLP-1 levels showed direct proportionality with several metabolic risk markers (i.e., BMI, leptin, insulin) over 109 men diagnosed with schizophrenia and on clozapine, while this association was not observed in women^131^. An exploratory analysis of genetic data of patients from the Clinical Antipsychotic Trials of Intervention Effectiveness (CATIE) trial showed that different haplotypes encoding for GLP-1R correlated with variable response rates to antipsychotic medications^132^.

#### Clinical studies

All 23 relevant clinical studies for this section focussed on the effects of GLP-1RAs on cardiometabolic parameters in people with schizophrenia-spectrum disorders on antipsychotics, apart from a secondary analysis investigating cognitive and mental health outcomes^133^ (Table 3, Extended Data Tables 3–4). This also applied to another 5 ongoing studies identified (Supplementary Information S3).

The four meta-analyses^134–137^ were successively updated to incorporate upcoming trials, so that the most recent^134^ included seven RCTs^136,138–143^. This meta-analysis showed that, over 398 antipsychotic-treated patients with schizophrenia followed up between 12 and 24 weeks, the GLP-1 RAs liraglutide and exenatide were superior to placebo for body weight, waist circumference, BMI, and blood pressure^134^. The meta-analysis by Wang and colleagues^137^ included an unpublished trial (NCT00845507^144^) that was not part of the more recent meta-analysis by Khaity and colleagues^134^. For this RCT, we identified a conference abstract that similarly reported a positive effect of exenatide on weight reduction and BMI^145^.

As mentioned, a secondary analysis of an RCT assessing the cardiometabolic effects of exenatide in 40 people with schizophrenia^139^, also looked at measures of cognition and psychosocial functioning (i.e., Brief Assessment of Cognition in Schizophrenia, Rey–Osterreith Complex Figure Test, Short-Form Health Survey, Personal and Social Performance Scale, Positive and Negative Syndrome Scale), but found no effect for this GLP-1RA compared to placebo over 3 months^133^. All other trials retrieved^138–143,145,146^ investigated cardiometabolic parameters and were included in the meta-analyses above^134,136,137^.

Three small cohort studies^147–149^ examined associations between GLP-1RAs use and metabolic changes in adults with a diagnosis of schizophrenia and co-morbid diabetes and/or obesity on antipsychotics. Of these, two studies (N = 16 and 46 respectively) observed a positive association between the use of exenatide, liraglutide, or dulaglutide and weight loss as well as HbA1c at 16 weeks^148^ and 1 year^149^, while for the smaller one (N = 7) this association was not significant^147^.

All case series/reports^150–154^ reported better metabolic outcomes in patients with comorbid severe mental illness and diabetes and/or obesity who were concomitantly treated with antipsychotics and GLP-1RAs. A qualitative sub-study of the trial by Whicher and colleagues^143^ over 17 overweight or obese patients with schizophrenia spectrum disorders found that most of the interviewee and their clinicians had had positive experiences regarding GLP-1RAs administrations.

Compared to other neuropsychiatric illnesses, most studies on the effects of GLP-1RAs in psychotic disorders seem to have focussed so far on their potential use to counteract the cardiometabolic side effects due to antipsychotic medications. While this is a key research area, we propose that further investigations should verify whether GLP-1RAs may also affect cognitive and behavioural symptoms seen in psychosis, as suggested by their potential to influence neurobiological (e.g., immune function) and neuropsychological (e.g., reward) mechanisms that are known to be disrupted in psychotic illness.

### Mood And Anxiety Disorders

#### Pre-clinical/mechanistic studies

Articles relevant to this section mainly addressed depressive and anxiety conditions, while only 2 pre-clinical studies investigated the effect of GLP-1RAs in bipolar disorder (Supplementary Information S5). In animal models of mania, liraglutide augmented the activity of the mood stabilisers sodium valproate^155^ and lithium^156^. This effect appeared to be mediated by antioxidant mechanisms involving GSK3 phosphorylation^155^, and it was also associated with a reduction of measures of memory impairment in mice^156^.

Several animal studies were found to be relevant for depression and anxiety^157–167^, although with conflicting results. Two studies on exenatide^161,165^ and one on liraglutide^161^ showed no effect of these GLP-1RAs on depression-like behaviour. One of these studies also failed to identify any change in anxiety-like behaviour^161^, while two further studies employing exendin-4 observed an anxiogenic effect following acute administration^157,168^. Intriguingly, one of these studies also showed that longer administrations can lead to a normalisation of anxiety and a dissociable improvement in depression-like behaviour^157^ – a pattern that resembles of the mechanisms of action of conventional antidepressants and that may be further suggestive of the activity of GLP-1RAs on the serotonin system^157^. Three further articles reported a beneficial effect of liraglutide on depression-^164^ as well as anxiety-like behaviour^163,166^ in rats/mice, possibly mediated by neuroprotective mechanisms in the hippocampus^163,166^, and improved cognitive function^164^. Similarly, both lixisenatide^162^ and dulaglutide^159^ administration led to positive changes in different paradigms of depression induced in mice.

Two studies investigated animal models of comorbid depression and epilepsy^158,160^ (Aygun 2021, DeSouza 2019): one showed that exendin-4 led to an increase in frequency of absence seizures as well as depressogenic and anxiogenic responses^158^, while the other saw a decrease of depression-like behaviour for liraglutide irrespective of concurrent use of the antiepileptic levetiracetam^160^. In an animal model of depression and diabetes however, exendin-4 led to antidepressant-like effects, which was associated with changes in microglial function^167^.

Finally, we identified 6 papers describing favourable associations between GLP-related molecules (i.e., geniposide, GLP2, puerarin) and reductions in depression-like behaviour^169–174^.

One study explored mechanistic associations between GLP-1 functioning and mood disorders in humans: a post-mortem investigation showed that, compared to healthy controls, patients who had been diagnosed with mood disorders had lower expression of the gene encoding for GLP-1R in the dorso-lateral prefrontal cortex and the hippocampus, while this association was not observed in the brain tissue of people with schizophrenia^175^. Further, a recent resting-state fMRI analysis of 18 women with obesity or PCOS randomised to either 16-week semaglutide or placebo showed no significant changes in brain regions associated with depression and suicidality^176^.

#### Clinical studies

We split this section between studies of GLP-1RAs in people with mood disorders and studies of mood symptoms in patients with other medical conditions taking GLP-1RAs (Tables 4-5, Extended Data Tables 5-6).

Only 4 clinical studies specifically examined GLP-1RAs in mood disorders (Table 4). One non-randomised open-label study, published over two separate articles, showed that 4-week liraglutide led to an improvement in a test of executive functioning (and possibly other cognitive measures)^177^ and related increase in fronto-striatal volumes^178^, partly moderated by BMI and insulin resistance changes, in 19 people diagnosed with bipolar disorder or major depression. A historical cohort investigation of 29 patients with comorbid mood disorder and obesity noted that liraglutide-induced weight loss over six months was not associated with changes in psychiatric symptoms, though less than half of the study population completed the study period^179^. Conversely, some case reports for exenatide^180^ and semaglutide^181^ described onset or relapse of depressive symptoms, which resolved when the GLP-1RAs were stopped^180,181^ and recurred on medication rechallenge^180^

In contrast, we found a larger amount of evidence over 26 studies assessing depressive symptoms in populations with comorbid physical health conditions undergoing GLP-1RA treatment (Table 5). A recent meta-analysis of mixed evidence (5 RCTs^51,182–185^ and 1 cohort study^186^) in 2,071 people with T2DM or Parkinson’s disease suggested antidepressant efficacy of the GLP-1RAs exenatide and liraglutide over 24-52 weeks^187^. The same finding had been reported by a prior larger meta-analysis (6,914 overweight/obese patients with T2DM) over 8 studies^183,184,188–193^, but only when the largest study that also included non-diabetic participants^192^ was excluded in a sensitivity analysis^194^. In fact, the omitted study was a pooled analysis of 5 RCTs^195–199^ of 5,325 patients with obesity followed for up to 3 years, which had shown that liraglutide was no different from placebo for depressive symptoms as scored on the PHQ-9, while also highlighting a small increased risk of suicidal behaviour^192^.

The above meta-analyses comprised all clinical trials we could retrieve with our search^51,182–185,188,190,195–199^. One of these trials also assessed anxiety symptoms and found no effect of liraglutide compared to placebo over 26 weeks in 80 patients with comorbid T2DM and obesity who had previously undergone bariatric surgery^185^.

Of the cohort studies already included above^186,189,191^, two report additional results of relevance. An early cohort study on a small number of diabetic patients (N = 138) saw reduced depression scores at 18 months in people exposed to exenatide compared to insulin independently from BMI changes^189^. This result was replicated in a similar but larger investigation (N = 1,735) comparing all available GLP-1RAs versus non-GLP-1RA antidiabetics, with this antidepressant association possibly correlating with changes in markers of systemic inflammation (i.e., high-sensitivity C-reactive protein)^191^. We also identified several further observational investigations. A recent and more extensive (N = 10,690 people with diabetes followed up over 6-7 years) historical cohort study observed a reduced association between GLP-1RA use compared to non-use for depressive and, more pronouncedly, anxiety disorders incidence, especially in women^32^. However, another study with similar design did not see any association between GLP-1RAs exposure and changes in new-onset depression or self-harm over 16,910 diabetic patients over approximately one year of follow up^200^. Two 10-year case-control studies over very large samples of people with diabetes (N = 360,205 and N = 73,869 respectively) equally observed no association between GLP-1RA use and incident depression^201,202^. Also, a small cross-sectional study of 36 women with PCOS noted no changes in depression scores associated with liraglutide use over 6 months^203^, while another reported worsening depressive symptoms, which correlated with higher perceived stress scores, in 43 diabetic and obese exenatide-users against non-users at 3 months^204^. Finally, following recent concerns by regulatory agencies regarding a potential increase in suicidal behaviour associated with GLP-1RAs^205^, we found one recent pharmacovigilance report showing 0.6% suicidal events among 41,236 safety reports for these medications^206^ and an emulated target trial of 86,418 older adults with T2DM that did not identify any difference in suicidal ideation or behaviour between GLP-1RAs and other antidiabetic medications over 1.5 years^207^. Instead, a historical cohort study of over 200,000 electronic health records observed a reduced association between semaglutide use and suicidality in both people with T2DM and obesity at one year^208^.

We did not identify any study that specifically addressed potential interactions between GLP-1RAs and commonly-used antidepressant medications.

Although several studies have investigated GLP-1RAs across mood and anxiety disorders, evidence appears mixed, as beneficial, harmful, and null effects have all been reported for depressive symptoms and suicidality. Furthermore, the evidence-base for the mechanisms possibly involved in the mood-regulating properties of these medications appear more tentative, and would benefit from a more in-depth assessment. At present, clear clinical recommendations regarding the safety of GLP-1RAs for people with pre-existing depression or suicidal behaviour cannot be made.

### Eating Disorders

#### Pre-clinical/mechanistic studies

We retrieved only a few pre-clinical and mechanistic articles relevant to GLP-1RAs for eating disorders (Supplementary Information S5). Higher GLP-1 levels inversely correlate with binge-like eating in animals^209,210^, and bingeing behaviour is associated with lower GLP-1R in the nucleus of the solitary tract (NST)^211^. The GLP-1RA exendin-4 reduced binge-like feeding in rats via action on -opioid receptors in the nucleus accumbens (NAc)^212^.

#### Clinical studies

Despite their thriving role in the treatment of obesity^213^, only 7 studies investigated the effects of GLP-1RAs in eating disorders (Table 6), including some on their psychopathology in comorbid obesity^214–216^ and others specifically in binge-eating disorder (BED)^216–219^, and we could not find any ongoing trial in this area. For a comprehensive review of the anti-obesity effects of GLP-1RAs, which is beyond the purpose of this article, see Chakhtoura (2023)^220^.

A single-arm trial showed that liraglutide reduced, from pre-exposure to 12-weeks post-exposure, the occurrences of uncontrolled and emotional eating in 36 women with obesity and polycystic ovary syndrome (PCOS)^215^. Similar results were observed in a later study for 69 obese adults using semaglutide^216^. However, a long-term exploratory RCT in 150 people with obesity found that differences in eating disorders’ psychopathology scores were not maintained at 52 weeks when liraglutide in combination with behavioural therapy was compared to behavioural therapy alone^214^.

An early RCT in 44 patients with obesity and sub-clinical binge eating showed that liraglutide was better than diet and exercise alone in reducing binge eating scores at 12 weeks^219^, but a later investigation of 27 adults with BED comparing liraglutide against placebo did not find any differences in the number of binging episodes over 17 weeks^217^. Another positive finding was seen in 60 patients with BED and T2DM when dulaglutide, which is not currently licensed for obesity, was compared to placebo at 12 weeks^221^. Over a longer follow-up of 180 days, a retrospective cohort study of semaglutide still observed lower scores in binge eating psychopathology than other anti-obesity medications in 48 patients with moderate to severe BED^218^.

Despite their established role in promoting weight loss, there is a paucity of research investigating the safety or efficacy of GLP-1RAs in people whose eating disorders have a psychopathological component (e.g., anorexia nervosa, bulimia nervosa) as conventionally defined by diagnostic manuals. While there may be some resistance to the conduction of clinical trials of pharmacological interventions in these clinical populations, the mechanistic profile of GLP-1RAs clearly suggests that these medications may play a role in the treatment of certain specific eating disorders, such as BED.

## Discussion

**Figure 1:**
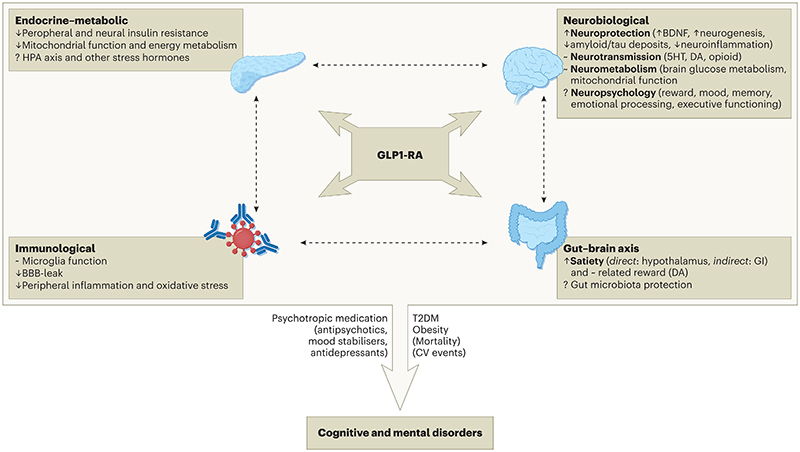
Established and putative modes of action of GLP-1RAs for cognitive and mental health disorders ***Legend***: BBB: blood-brain barrier; BDNF: brain-derived neurotrophic factor; DA: dopamine; GI: gastrointestinal; HPA: hypothalamus-pituitary axis; ↑: increase; ↓: decrease; ~: regulates;?: uncertain. The potential usefulness of GLP-1RAs in psychiatric disorders may be underpinned by their multimodal actions in the CNS and beyond: decreasing inflammation and oxidative stress, reducing neural insulin resistance, modulating neural metabolism and microglial function, as well as regulating key neurotransmitter pathways. In addition, the cardiometabolic benefits of these agents could lead to improved morbidity and mortality outcomes in this patient population. GLP1-RA effects on higher-order neuropsychological processes, on stress responses, or on the gut microbiome remain to be explored.

**Figure 2:**
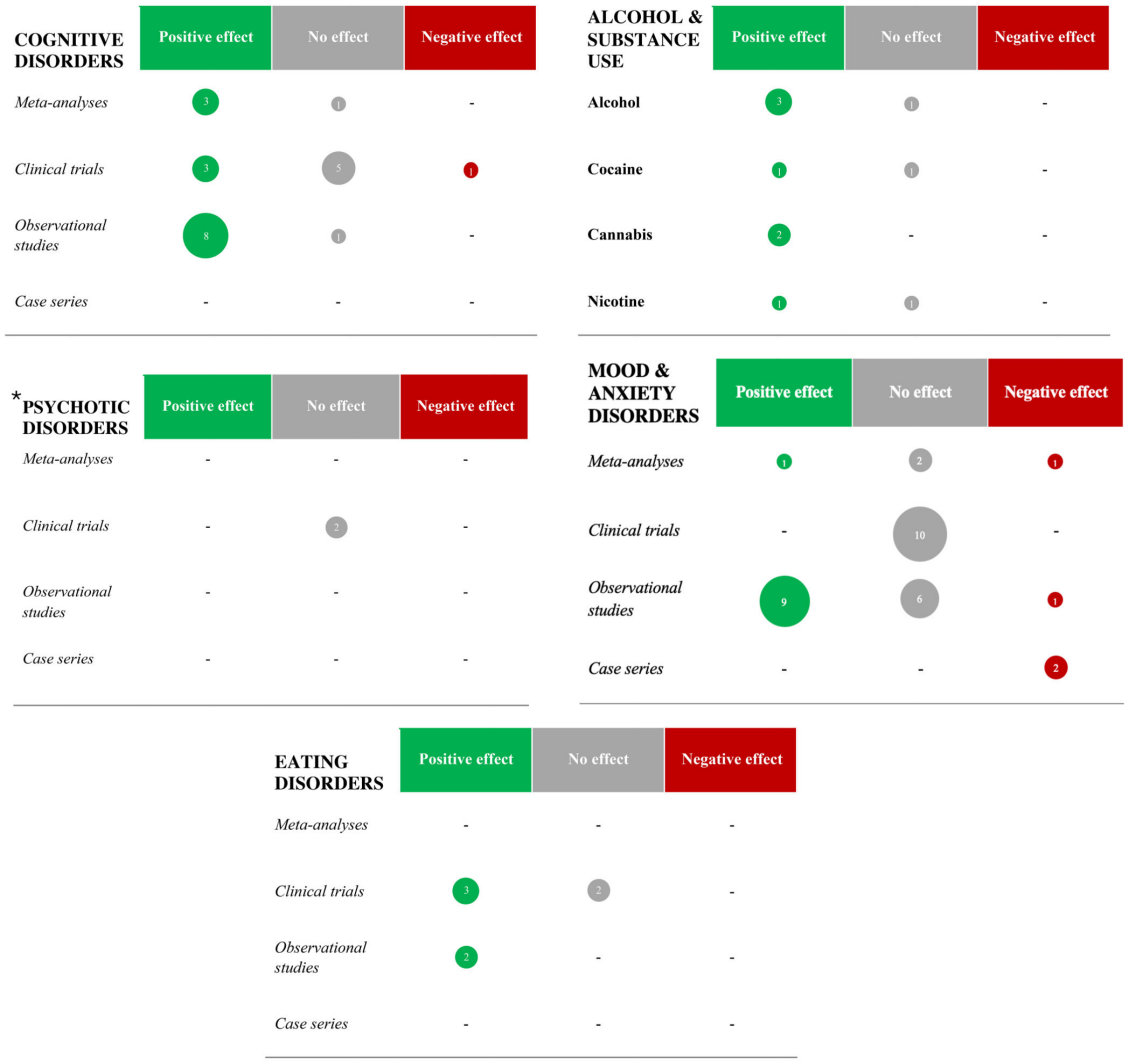
Summary of clinical studies of GLP-1RAs for cognitive and mental disorders ***Legend***: Green: positive effect/association; grey: no effect/association; red: negative effect/association; the area of each circle is proportional to the number of studies. *Does not include studies of metabolic effects of GLP-1RAs in people with psychotic disorders, which would not be in line with the psychiatric outcomes reported for all other disorder

In this article, we reviewed pre-clinical/mechanistic (*in vitro*, in animal, and in human) and clinical studies, leveraging potential translational aspects, on GLP-1RAs across a variety of cognitive and mental health disorders. Overall, we identified 280 pre-clinical/mechanistic (Figure 1 and Supplementary Information S4 and S5) and 96 clinical studies (Tables 1-6, Figure 2, Extended Data Figure 1, Extended Data Tables 1-6, and Supplementary Information S2), with a clear trend of growing relevant literature over the past few years as the use of these medications becomes more widespread and their indications expand far beyond the initial intentions of the manufacturers^222^. Some key messages and common themes emerge.

First, there is supporting evidence for the safety of GLP-1RAs across the board of cognitive and mental disorders, as we retrieved very few studies^44,158,180,181,192,204^ suggesting worse neuropsychiatric outcomes associated with these medications (Tables 1-6, Figure 2, Extended Data Tables 1-6, and Supplementary Information S2). A recent meta-analysis of 31 RCTs including 84,713 patients comparing any GLP-1RA against placebo found no difference in the incidence of adverse neuropsychiatric events over >1 year^223^, and several pharmacovigilance studies published over the last year have been in line with such results^224–228^ (Supplementary Information S2). Publication bias and poor recording of adverse events, which is common in clinical trials, may however explain such paucity of safety signals. In July 2023, the European Medicines Agency^205^ and the UK Medicines and Healthcare products Regulatory Agency (MHRA)^229^ started a review of these medications’ safety following reports of worsening mood and suicidal behaviour observed in GLP-1RA users. In the USA, prescribing information for all medications licensed for obesity that act on the central nervous system, including the GLP-1RAs liraglutide 3mg (Saxenda®) and semaglutide 2.4mg (Wegovy®), must include the recommendation of monitoring for depression and suicidal ideation^230^. This however does not apply to other GLP-1RAs approved for the treatment of T2DM, including the same liraglutide (Victoza®) and semaglutide (Ozempic® or Rybelsus®) at lower dosages, prompting several stakeholders to request an updated guidance^231^ and more caution in media enthusiasm^232^. Indeed, the history of anti-obesity medications has been marked by several failures due to serious adverse events, such as suicidality, observed only after their usage had become extensive^220,233^ – a well-known example being the one that led to the withdrawal of the endocannabinoid inverse agonist, rimonabant^234^. Many have advocated that the associations between low mood, suicidal behaviour, and anti-obesity drugs such as GLP-1RAs are confounded by the pre-existing higher prevalence of neuropsychiatric disorders observed in people living with obesity compared to the general population^235^. More recently, the EMA Pharmacovigilance Risk Assessment Committee has concluded that the available evidence does not presently support a causal association between GLP-1RAs and suicidality^236^. Overall, as GLP-1RAs become increasingly prescribed, further pharmacovigilance studies are warranted.

Second, considering evidence from clinical studies as informed by pre-clinical and mechanistic research, a putative benefit of GLP-1RAs on cognitive disorders (mediated by several neuroprotective mechanisms, especially anti-inflammatory effects) (Figure 1, Table 1, Extended Data Tables 1-2, Supplementary Information S4) and substance use disorders (via modulation of dopaminergic pathways of reward, impulse-control, and decision-making) (Figure 1, Table 2, Supplementary Information S5) seems more likely, while any effect on psychotic, mood, and anxiety disorders appears less consistent and in need of further investigation. This would be in line with a recent propensity-score matched cohort study by our laboratory, which observed that semaglutide was associated with reduced cognitive deficit and nicotine misuse when compared against three other antidiabetic medications^226^. It is also possible that GLP-1RAs may have a therapeutic effect across traditional diagnostic categories. For example, inflammation is known to play a role in at least a subset of depressive^237^ and psychotic disorders^238^, therefore it is conceivable that the use of GLP-1RAs may be beneficial in these patients’ groups – although no studies have specifically assessed these mechanistic aspects in relation to psychopathology in humans thus far. Clinically, GLP-1RAs could lead to an improvement in cognitive function, which is often found to be impaired across several conditions such as psychosis^239^ and mood disorders^240^, eventually leading to an overall benefit as observed in some of the included studies (Tables 4-5). This notion is speculative at present, since no change in cognition was observed in one small RCT of exenatide in schizophrenia^241^, while a positive cognitive effect of liraglutide was only seen in an even smaller non-randomised open-label investigation of people with either depressive or bipolar disorders^177,178^. Notably, an ongoing RCT investigating the effects of semaglutide on pre-treatment cognitive dysfunction in patients with major depression may provide useful insights in this regard (NCT04466345^242^, Supplementary Information S3). On the other hand, the plausible actions of GLP-1RAs on several reward domains may require more nuanced interpretation. Alcohol and other substance use disorders may well benefit from the effects of GLP-1RAs on dopamine and opioid pathways that are dysregulated in addiction^243^, as seen in some of the studies we identified, and the same could also apply to other under-investigated disorders with similar underlying dysfunctions (e.g., gambling disorder). Conversely, people who already present with significant anhedonia, for instance in the context of a depressive illness, may see their symptoms worsening when on GLP-1RAs – which could elucidate some of the studies reporting negative effects associated with these medications in mood and anxiety disorders. As hinted above, this predicament could be disentangled via studies that include a mechanistic assessment of biomarkers predicting response *vs* harm following GLP-1RAs administration^191^.

Any potential transdiagnostic benefit of GLP-1RAs may be amplified by their established effects on cardiovascular and metabolic morbidity and mortality^21,22^, which are known to be raised in several cognitive and mental health disorders^244,245^. Indeed, an important issue for the potential cognitive and mental health effects of GLP-1RAs, which our review cannot fully address, is whether these medications provide symptomatic relief only via their well-established cardiometabolic benefits, or by directly targeting physiopathological mechanisms behind cognitive and mental symptoms. Only a minority of studies i.e., 4 in Parkinson’s disease^51–54^, 5 in substance use disorders^111,113,115–117^, 1 in psychotic disorders^143^, and 2 in mood disorders^177,178,181^ assessed the cognitive and mental health effects of GLP-1RAs in non-diabetic, non-obese populations. As research on GLP-1RAs expands in the cognitive and mental health area, we may be able to distinguish between direct effects on cognitive and mental health outcomes and effects that are mediated by GLP-1RAs’ actions on cardiovascular and metabolic outcomes. The numerous ongoing/planned studies reported in Supplementary Information S3 will likely provide more clarity in this regard.

On this note, we also observed a lack of studies examining possible interactions between psychotropic medications and GLP-1RAs – perhaps due to the novelty of the latter. Nevertheless, numerous ongoing trials are investigating the cardiometabolic effects of GLP-1RAs in patients with mental illness, especially for those on antipsychotics (Supplementary Information S3) – such research should therefore address the abovementioned knowledge gap.

Third, we only found a few studies on GLP-1RAs in eating disorders and their psychopathology (Table 6, Figure 2). To our knowledge, no study assessed the potential of abuse of these medications anecdotally reported in anorexia or bulimia nervosa, which would require further investigation. Interestingly, obesity, for whose treatment GLP-1RAs are approved and validated^1,213^, is not classified under mental and behavioural disorders, and in some countries such as the UK it is not even formally recognised as a disease^246^. In this context, we note that the remarkable effects of GLP-1RAs in achieving weight loss may fail to be maintained over the long term once medications are stopped^247^. Some have argued that obesity is a severely under-treated condition, despite its high prevalence, comorbidity with many physical and mental health disorders, and associated mortality and societal cost^246^. Although several psychological factors (e.g., deficit in impulse-control) are known to play a major role in the pathophysiology of obesity^248^, we here raise the issue of disparity in the provision of psychiatric care for the treatment of obesity compared to other eating disorders, which are predominantly treated by psychiatrists, and advocate for the importance of a multidisciplinary, integrated approach to weight management.

Fourth, a significant issue that is often raised is whether any GLP-1RAs can indeed penetrate the blood-brain barrier and therefore express any neurobiological activity in the CNS, which would result in cognitive or behavioural changes. Some studies in rodents showed that exendin-4^249^, liraglutide^250^, and semaglutide^5^ did not cross the BBB but instead interacted with the brain through the circumventricular organs. However, other investigations have suggested that several GLP-1RAs may cross the BBB via passive diffusion^251^, GLP-1R-mediated uptake mechanism^252^, or adsorption transcytosis^253^, although different compounds may present with variable degrees of brain penetrance^254^. Overall, the extent to which GLP-1RAs cross the BBB remains uncertain in pre-clinical studies^4^, and further discrepancies are expected in translating these data from animals to humans. Additionally, some putative effects of GLP-1RAs on cognitive and mental health symptoms may not require direct activity in the CNS, but rather be mediated by the actions that these medications express in the periphery across immune, endocrine-metabolic, and gut-brain axis mechanisms – see Figure 1. Finally, another layer of complexity is added when considering the evidence of leaky BBB across several neuropsychiatric disorders^255^, which could further increase the brain penetrance of GLP-1RAs administered to people with such illnesses.

### Limitations

In this paper, our methodology was systematic in nature (Supplementary Information S1) since we sought to maximise the comprehensiveness of our search whilst providing a balanced overview of available literature. Limitations of this approach however include the lack of quantitative analysis and of a structured assessment of the quality of studies and certainty of evidence, which were beyond the scope of this descriptive work. Further, we did not use operationalised criteria (e.g., Diagnostic and Statistical Manual 5^th^ edition) to define the populations of interest because these would not be applicable across animal and human studies, but relied on the definitions provided by the individual articles. Finally, sex assigned at birth was not assessed in this review work. These limitations can be more appropriately addressed in future systematic reviews with meta-analyses.

### Conclusions

In conclusion, some have argued that GLP-1RAs have the potential to transform medicine and society as we know it^256^, which will undoubtedly have a profound impact on psychiatric practice. High costs, as well as tolerability issues, remain significant barriers to a more wide-ranging prescribing of these drugs^1^. The pharmaceutical industry is developing newer and potentially cheaper or more effective molecules that target GLP-1 and associated pathways (e.g., the so-called dual- and triple-agonists tirzepatide, retatutride, and orfoglipon)^257,258^. The promise of GLP-1RAs could materialise for several cognitive and mental health disorders. Still, caution is required because the adoption of general medical treatments into psychiatry (for example, insulin therapy) has sometimes led to deleterious consequences for patients. Conscious of the importance of all the above, we argue for the need of and inquisitive mechanistic and clinically applied research to inform stakeholders about the potential benefits and harms of GLP-1RAs. This should include a more accurate, scientifically-sound, and perhaps sober guidance of the communication between the media and the public.

## Methods

This review did not require ethical approval, and a protocol was not pre-registered. We conducted a search of the literature on 20^th^ November 2023 via Ovid SP® of PubMed/MEDLINE, Embase, Cochrane CENTRAL, PsycInfo databases from inception, updated with serial manual searches until 13^th^ July 2024. ClinicalTrials.gov and the WHO portal were also reviewed for ongoing or unpublished studies. The broad search algorithm combined index terms and free-text words for all GLP1-RAs, with no restriction to study language, design (including both individual studies and their meta-analyses), setting, comparator, and outcome of interest to maximise the comprehensiveness of the evidence synthesis. The web-based software, Covidence, for semi-automated text mining, and extensive forward/backward searching were employed to support with de-duplicating and screening of records to only include studies relevant to cognitive and mental health disorders. Two researchers (AG, OD) independently screened titles and abstracts for relevance, assessed the full texts for eligibility, and extracted relevant data; disagreements were discussed with a third author (RDG) and resolved by consensus to data validation. Studies were divided between pre-clinical/mechanistic evidence and clinical evidence; both were fully described so that the former could support the interpretation of the latter. We used a systematic approach to literature searching and data extraction to increase the transparency of the data reported, but no statistical methods were used with the data collected.

## Extended Data

**Extended Data Table 1:** Clinical studies of GLP-1RAs for cognitive disorders, clinical trials.

Study ID	Design	Population	Intervention/Exposure	Comparison	Followup	Outcomes	Major findings	
**Clinical trials**								
Athauda 2017	RCT	60 adults PD	Exenatide	Placebo	1.2 years	MDS-UPDRS part I	MD = -3.5 95% CI = -6.7. -0.3 (p=0.0318)	+
Aviles-Olmos 2014	RCT (open label)	44 adults PD	Exenatide	Usual PD medication	2 years	MDS-UPDRS part I	Liraglutide: 2.0±4.2, 95% Cl = 0.0,4.0 Control: 5.1±5.5, 95% Cl = 2.8, 7.4 (p=0.049)	=
Cheng 2022	RCT	36 patients T2DM	Liraglutide	Dapagliflozin, Acarbose	4 months	MMSE, MoCA	“Not markedly changed by any of the three treatments between baseline and week 16”	=
Cukierman- Yaffe 2020	RCT	8,828 adults T2DM	Dulaglutide	Placebo	5.4 years	MoCA, DSST	HR = 0.86 95% Cl = 0.79, 0.95 (p=0.00J8)	+
Hogg 2022	RCT	63 adults PD	Liraglutide	Placebo	1 year	MDS-UPDRS part I	Liraglutide: -0.9±4.7, Placebo: 0.5±4.4 (p=0.29)	=
Husain 2019	RCT	3,183 adults T2DM	Semaglutide	Placebo	13 years	Rate of dementia (SMQ)	Outcomes only reported in pooled analysis by Norgaard et al. 2022 (Semaglutide: 0, Placebo: 0.96)	NA
Li 2021	RCT	47 adults T2DM	Liraglutide	Other antidiabetic	3 months	MMSE	Liraglutide: 28.96±1.00 vs Qther antidiabetic: 27.48±1.73 (p=0.040)	+
Marso 2016a	RCT	9,340 adults T2DM	Liraglutide	Placebo	33 years	Rate of dementia (SMQ)	Outcomes only reported in pooled analysis by Norgaard et al. 2022 (Liraglutide: 0.67, Placebo: 1.41)	NA
Marso 2016b	RCT	3,297 adults T2DM	Semaglutide	Placebo	2.1 years	Rate of dementia (SMQ)	Outcomes only reported in pooled analysis by Norgaard et al. 2022 (Semaglutide: 0.88, Placebo: 1.47)	NA
Meissner 2024	RCT	156 adults PD	Lixisenatide	Placebo	1 year	MDS-UPDRS part 1	MD= -0.64 95% Cl =-1.83,0.55	=
Wang 2020	RCT	60 patients T2DM and post-stroke MCI	Sitagliptin	Liraglutide	6 months	MMSE, MoCA	Sitagliptin > Liraglutide (p<0.01)	=
Zhang 2019	RCT	19 adults obesity and any diabetes	Exenatide, Liraglutide	Pre-treatment baseline	3 months	MoCA	Baseline: 26.6±2.4, after treatment: 27.9±1.9 (p=0.0)4)	+

***Legend***: + : positive effect; = : no effect; - : negative effect. Values are mean±SD unless otherwise specified. Study ID reports the first author and year only.

DSST: Digital Symbol substituation Tesy; HR: Hazard Ratio; MCI: Mild Cognitive Impairement; MD: Mean Difference; MDS-UPDRS: Movement Disorders Society Unified Parkinson’s Disease Rating Scale; MMSE: Mini-Mental State Examination; MoCA: Montreal Cognitive Assessment; NA: Not Available; RCT: Randomised Contrlled Trail; PD: Parkinson’s Disease; SMQ: Short-Memory Questionnaire; T2DM: Type 2 Diabetes Mellitus.

**Extended Data Table 2:** Clinical studies of GLP-1RAs for cognitive disorders, observational studies.

Study ID	Design	Population	Intervention/Exposure	Comparison	Follow- up	Outcomes	Major findings	
**Cohort studies**								
Secnik 2020	Prospective cohort	133,318 adults any diabetes	Any GLP-lRAs	Nonusers of GLP-lRAs	14 years	Risk of any dementia	**HR =** 0.51 95% Cl = 0.41,0.63 (p<0.001)	4-
Zhou 2021	Historical cohort	342,608 patients T2DM	Exenatide	Nonusers of GLP-lRAs	5 years	Risk of AD	OR = 0.98 95% Cl = 0.96, 0.99 (p<0.001)	+
**Case-control studies**								
Akimoto 2020	Case-control	66,085 older adults T2DM (1,250 concomitant AD)	GLPl-RAs (Dulaglutide, Exenatide, Liraglutide) + Metformin	Metformin-only	14 years	Risk of AD	Exenatide: aOR = 0.22 95% Cl = 0.11,0.37 (p=0.001) Liraglutide: aOR = 0.36 95% Cl =0.19, 0.62 (p<0.001) Dulaglutide: aOR = 0.39 95% Cl =0.17,0.77 (p=0.014)	+
Bohlken 2018	Case-control	8,276 adults T2DM and any dementia, 8,276 adults T2DM without dementia	Patients with dementia (1.7% on any GLP-lRAs)	Patients without dementia (2.1 % on any GLP-lRAs)	5 years	Risk of any dementia	OR = 0.90 95%CI = 0.70, 1.15 (p=0.387)	=
Ndigaard 2022	Nested case-control	120,054 adults T2DM	Any GLPl-RAs	Other antidiabetic	7.4 years	Risk of any dementia	**HR =** 0.89 95%CI = 0.86, 0.93	+
Wium- Andersen **2019**	Nested case-control	58,095 adults T2DM	Any GLPl-RAs	Nonusers of GLP-lRAs	7.2 years	Risk of any dementia	OR = 0.58 95% CI = 0.50,0.67	+
**Cross-sectional studies**								
Longo 2023	Cross- sectional	154 patients T2DM	GLP-1 RAs + Metformin	Metformin-only	>12 months	MoCA	**GLP-IRA + metformin: 26.5** (IQR 23.0 - 29.0), metformin only: 19.0 (IQR 17.0-24.2) (d<0.001)	+

***Legend***: + : positive effect; = : no effect; - : negative effect. Values are mean±SD unless otherwise specified. Study ID reports the first author and year only.

AD: Alzhimer’s; Disease; aOR: Adjusted Odds Ratio; GLPI-RA: Glucago-Like Peptide-1 Recceptor Agonist; HR: Hazard Ratio; IQR: Interquartile Range; MMSE: Mini-Mental State Examination; MoCA: Montreal Cognitive Assessment; OR: Odds Ratio; T2DM: Type 2 Diabetes Mellitus.

**Extended Data Table 3:** Clinical studies of GLP-1RAs for psychotic disorders, clinical trials

Study ID	Design	Population	Inter vend on/Exposure	Comparison		Follow-up	Outcomes	Major Findings
**Clinical trials**								
Eriksson 2019	RCT - 2° analysis of lsh0y 2017a	40 adults obesity, nondiabetic, on antipsychotics	Exenatide	Placebo	3 months	Bone turnover markers (CTX, PINP) and BMD	No significant changes	=
Ishey 2017a	RCT	40 adults schizophrenia- spectrum, obesity, nondiabetic, on antipsychotics	Exenatide	Placebo	3 months	Body weight (kg)	Exenatide: -2.2±3.3, Placebo: - 2.2±4.4 (p=0.98)	=
lsh0y 2017b	RCT - 2° analysis of lsh0y 2017a	40 adults schizophrenia- spectrum, obesity, nondiabetic, on antipsychotics	Exenatide	Placebo	3 months	Cognition (BACS)	Exenatide baseline: 0.05±0.73, after treatment: -0.29±0.76, Placebo baseline: -0.05±0.78, after treatment: 0.16±0.72 (p=0.77)	=
Larsen 2017	RCT	103 adults schizophreniaspectrum, on Clozapine or Olanzapine	Liraglutide	Placebo	4 months	Body weight (kg)	MD = -5.3 95% CI = -7.0, -3.7 (p<0.001)	+
Maagensen 2021	RCT - 2° analysis of Larsen 2017	72 adults schizophreniaspectrum, on Clozapine or Olanzapine	Liraglutide	Placebo	4 months	Bone turnover markers (CTX, PINP)	No significant changes	+
Patino 2015	RCT	60 adults major mood or psychotic disorders, on Olanzapine	Exenatide	Placebo	4 months	Body weight (lbs)	MD = -7.9 (p=0.02)	+
Siskind 2017	RCT	28 adults schizophrenia, obesity, on Clozapine	Exenatide	Usual care	6 months	Body weight (kg)	MD =-4.16±5.99 (p=0.015)	+
Siskind 2020	RCT	27 adults schizophrenia, obesity, with or without T2DM, on Clozapine	Exenatide (after 6 months of treatment)	Usual care	1-year followup from Siskind 2017	Body weight (kg)	MD = 8.28 ± 2.03(SE) (p<0.001)	=
Svensson 2019	RCT	88 adults schizophreniaspectrum, on Clozapine or Olanzapine	Liraglutide (after 4 months of treatment)	Placebo	1 -year followup from Larsen 2017	Body weight (kg)	MD = 1.5 95% Cl = -1.8,4.7(p=0.38)	=
Whicher 2021	RCT	47 adults psychotic disorders, on antipsychotics	Liraglutide	Placebo	6 months	Body weight (kg)	MD = -6.0 95% Cl = -10.8,-1.36 (p=0.015)	+
						BPRS	MD = -6.3 95% Cl = -13.6, 1.0 (p=0.088)	=

***Legend***: + : positive effect; = : no effect; - : negative effect. Values are mean±SD unless otherwise specified. Study ID reports the first author and year only.

BACS: Brief Assessment of cognition in Schizophrenia; BMD: Bone Mineral Density; BPRS: Brief Psychiatric Rating Scale; CTX: Collagen Type 1 C-Telopetide; GLP1-RA: Glucagone-Like Peptide-1 Receptor Agonist; MD: Mean Difference; PINP: procollagen Type 1 N-terminal Pro-peptide; RCT: Randomised Controlled Trail; SE: Standard Error.

**Extended Data Table 4:** Clinical studies of GLP-1RAs for psychotic disorders, observational studies

Study ID	DesignPopulation			Intervention/ Exposure	Comparison	Follow-up	Outcomes	Major Findings
**Cohort Studies**								
Ando 2018	Prospect ive cohort	5 adults schizophrenia, diabetes, on antipsychotics	Liraglutide or Exenatide or both	Pre-treatment baseline	1 year	Body weight (kg)	-3.7 (range -9.6 to 3.5) (p=0.14)	**=**
Lee 2021	Historical cohort	16 adults obesity, on antipsychotics	Liraglutide	Pre-treatment baseline	4 months	HbAlc Body weight (kg)	-1.2 (range 0.1 to 3.4) (p=0.089) MD: -4.3 95% CI = -6.6, -2.0 (p<0.01)	**+**
Perlis 2020	Historical cohort	46 adults diabetes, on antipsychotics	Liraglutide or Exenatide or Dulaglutide	Other antidiabetic	1 year	Body weight (kg)	GLPl-RAs: -7.07 ± 2.62(SE), Control: 1.93 ± 1.14(SE) (p<0.05)	**+**
						HbAlc	GLPl-RAs: -1.26 ± 0.17(SE), Control: -1.47 ± 0.45(SE)	**=**
**Case series**								
Ishoy 2013	Case study	**1 adult** schizophrenia. T2DM, and obesity	Liraglutide	Pre-treatment baseline	2 years	Body weight (kg)	-7.7	**+**
						HbAlc	−4.0	**+**
Noda 2022	Case study	1 adult schizophrenia, T2DM, and obesity	Semaglutide (replaced Dulaglutide)	Dulaglutide	6 months	Body weight HbAlc	“Semaglutide was more effective than dulaglutide in reducing and maintaining HbAlc and body weight for 6 months after initiation of the drug.”	**+**
Prasad 2023	Case series	12 adults obesity on antipsychotics	Semaglutide	Pre-treatment baseline	1 year	Body weight (kg)	MD=−8.67±9 (p=0.04)	**+**
Siskind 2016	Case study	1 adult schizophrenia, T2DM, and obesity	Exenatide	Pre-treatment baseline	6 months	BMI (kg/m^2^)	−10	**+**
						Waist circumference (cm)	−28	**+**
Zhang 2022	Case study	1 adult schizophrenia. T2DM. and obesity	Liraglutide	Baseline	2 years	BMI (kg/m^2^)	−2.87	**+**
						**HbAlc**	**-6.3**	**+**
**Qualitative studies**								
Barnard- Kelly 2022	Qualitative sub-study of RCT	17 adults schizophrenia spectrum, overweight or obesity	Liraglutide	=	6 months	Qualitative interviews (5- 37min)	**“Most of those who completed the trial reported no challenges in** the timing of or administering the injections. Key themes included despondency regarding prior medication-associated weight gain, quality of life impact of weight loss, and practical aspects of participation including materials received and clinic attendance”.	**+**

***Legend***: + : positive effect; = : no effect; - : negative effect. Values are mean±SD unless otherwise specified. Study ID reports the first author and year only.

BMI: Body Mass Index; GLPI: Glucagon-Like Peptide-1 Receptor Agonist; AbA1c: Haemoglobin A1c; MD: Mean Difference; RCT:Randomized Controlled Trail; SE: Standard Error; T2DM: Type 2 Diabetes Mellitus

**Extended Data Table 5:** Clinical studies of GLP-1RAs for mood and anxiety disorders, effects on depressive symptoms in patients with other comorbidities, clinical trials

Study ID	Design	Population	htervention/Exposure	Comparison	Follow up	Outcomes	Major Findings	
**Clinical trials**								
Astrup **2009**	Open-label RCT	564 adults obesity	Liraglutide	Placebo. Orlistat	20 weeks	NA	NA	NA
Best 2011	RCT	491 adults T2DM	Exenatide + Metformin	Pioglitazone, Sitagliptin + Metformin	**26** weeks	PGWB (depression subscale)	Exenatide: 3.84 ± 1,33(SE) (95% Cl = 1.22, 6.45) Pioglitazone: 3.80 ± L30(SE) (95% CI = 1.24, 6.37) Sitagliptin: 3.73 ± 1.36(SE) (95% CI = 1.06, 6.40)	=
Blackman 2016	RCT	359 adults obesity and obstructive sleep apnoea	Liraglutide	Placebo	**32** weeks	PHQ-9, CSSRS	“No notable differences between liraglutide and placebo were observed during mental health evaluations with PHQ-9 and CSSRS”	=
Bode 2010	RCT	732 adults T2DM	Liraglutide	Glimepiride	1 year	HRQoL (depression subscale)	“No significant differences in depression subscale (p=0.154 to 0.339)”	=
Davies 2015	RCT	846 adults T2DM and obesity	Liraglutide	Placebo	**56** weeks	NA	NA	NA
de Wit 2014	Open-label RCT	50 adults T2DM and >4% weight gain during shortterm insulin therapy	Liraglutide	Insulin	26 weeks	BDI-II	“No change (p = 0.46)”	=
de Wit 2016	Open-label single-arm extension of RCT (de Wit 2014)	18 adults T2DM on stable insulin therapy	Liraglutide	Insulin + Liraglutide	26 weeks	BDI-II	“No change (p>0.05)”	=
Idris 2013	Non-randomised controlled trial	8 adults T2DM. obesity, and excessive daytime sleepiness	Exenatide	Placebo	**22** weeks	BDI	“Non-significant reduction between placebo and exenatide, which persisted after adjustment for HbAlc and weight change”	=
Miras 2019	RCT	80adultsT2DM and obesity undergone metabolic surgery	Liraglutide	Placebo	**26** weeks	HADS (depression subscale)	MD = -0.3 95% Cl-1.8, 1.3 (p=0.741)	=
Pi-Sunyer 2015	RCT	3,731 adults obesity	Liraglutide	Placebo	56 weeks	PHQ-9	“No clinically relevant differences for any assessments of mental health”	=
Wadden 2013	RCT	422 adults obesity	Liraglutide	Placebo	**56** weeks	PHQ-9	Liraglutide: -1.2±2.2, Placebo: 1.3±2.3	=

***Legend***: + : positive effect; = : no effect; - : negative effect. Values are mean±SD unless otherwise specified. Study ID reports the first author and year only.

BDI-II: Beck’s Depression Inventory-II; CSSRS: Columbia Suicide Severity Rating Scale; GLP1-RA: Glucagon-Like Peptide-1 Receptor Agonist; HADS: Hospital Anxiety and Depression Scale; HRQ-oL: Health-Realted Quality of Life; MD: Mean Difference; NA: Not Available; PGWB: Psychological Gendral Well-Being; PHQ-9: Patient Health Questionnaire; RCT: Randomized Controlled Trail; T2DM: Type 2 Diabtes Mellitus.

**Extended Data Table 6:** Clinical studies of GLP-1RAs for mood and anxiety disorders, effects on depressive symptoms in patients with other comorbidities, observational studies.

Study ID	Design	Population	Intervention/Exposure	Comparison	Follow-up	Outcomes	Major Findings	
**Cohort studies**								
Gamble 2018	Historical cohort	16,910 adults T2DM	GLP-IRAs	Sulfonylureas	1.1 years	Risk of new-onset depression or self-harm	HR = 1.25 95% CI = 0.63,2.50	=
Grant 2011	Prospective cohort	138 adults T2DM	Exenatide	Insulin	6 months	HADS	GLP-IRA: 12±4. Insulin: 17±4 (p = 0.041)	+
Moulton 2016	Prospective cohort	1,735 aduIts T2DM	GLP-IRAs, DPP-41 (incretins)	Non-incretin glucose- lowering agents	1 year	PHQ-9	Incretins: -2.68±5.70, Non-incretins - 0.17±4.70 (p=0.017)	+
Reaney 2013	Prospective cohort	2,388 adults T2DM	Exenatide	Insulin	2 years	HADS (depression subscale)	Exenatide: 5.44±4.09, Insulin: 6.04±4.35	=
Tang 2024	Emulated target trial	43,614 older adults T2DM	GLPl-RAs	SGLT-21	1.54-1.64 years	Incidence of suicidal ideation/behaviour	aHR = 1.07 95% CI = 0.80, 1.45	=
		42.804 older adults T2DM	GLPl-RAs	DPP-41	1.54-1.64 years	Incidence of suicidal ideation/behaviour	aHR = 0.94 95% CI = 0.71, 1.24	=
Tsai 2022	Historical cohort	53,456 adults any diabetes	Dulaglutide, Exenatide, Liraglutide	Nonusers of GLP- IRAs	7 years	Incidence of anxiety and/or depression	aHR =0.8 95% CI = 0.67,0.95 (p <0.01)	+
Wang 2024c	Historical cohort	240,618 adults overweight or obesity	Semaglutide	Non-GLPl-RA antiobesity medications	6 months	Incident suicidal ideation	HR = 0.27 95% CI = 0.20,0.36	+
		1,589,855 adults **T2DM**	Semaglutide	Non-GLPl-RA anti**obesity medications**	6 months	Incident suicidal ideation	HR = 0.36 **95% CI = 0.25, 0.53**	+
**Case-control studies**								
Kessing 2020	Nested case-control	360,205 adults T2DM	Exenatide, Liraglutide	Nonusers of GLP- IRAs	10 years	Incident depression or use of antidepressant	Exenatide: HR = 0.93 95% CI = 0.75, 1.15 (p=0.503) Liraglutide: HR = 1.10 95% CI = 1.00, 1.21 (p=0.048)	=/+
Wium Andersen 2022	Nested case-control	232,707 adults T2DM	GLP-lRAs	Nonusers of GLP- IRAs	10 years	Incidence of depression	**OR** = 0.77 95% CI = 0.71,0.84	+
**Cross-sectional studies**								
Eren- Yazicloglu 2021	Cross-sectional study	43 adults T2DM and obesity	Exenatide	Nonusers of Exenatide	3 months	PHQ-9	Exenatide: 9.70±4.92, Nonusers: 6.70±4.66 (p=0.026)	=
Ruggiero 2024	Pharmacovigilance study	41.236 safety reports	Any GLPl-RAs	/	From 1 January 2018to 10 July 2023	Incidence of suicidal events	N = 230 (0.6%) reported at least one suicidal event, including suicidal ideation (65.3%) and suicide attempt (19.5%)	NA
Kahal 2019	Cross-sectional study	36 adult women with or without PCOS	Liraglutide in PCOS subjects	Liraglutide in age and weight-matched controls	6 months	Depression (CES-D score ≥ 16)	PCOS baseline: 32%, after treatment: 26% (p=0.72), non-PCOS baseline: 29%, after treatment: 18% (p=0.42)	=

***Legend***: + : positive effect; = : no effect; - : negative effect. Values are mean±SD unless otherwise specified. Study ID reports the first author and year only.

aHR: Adjusted Hazard Ratio; CES-D: Center of Epidemiologic Studies Depression Scale; Dpp-4I: Dipeptidly Peptidase-4 Inhibitory; GLPI-RA: Glucagon-Like Peptide-1 Receptor Agonist; HADS: Hospital Anxiety and Depression Scale; HR: Hazard Ratio; NA: Not Availablel; OR: Odds Ratio; PCOS: Polycystic Ovary Syndrom; PHQ-9:Patient Health Questionnaire; SGLT:2I: sodium-glucose cotansporter-2 inhibitors; T2DM: Type 2 Diabtes Mellitus.

## Supplementary Material

Supplementary Information

## Figures and Tables

**Table 1:** Clinical studies of GLP-1RAs for cognitive disorders, meta-analysis

Study ID	Design	Population	Intervention/Exposure	Comparison	Follow-up	Outcomes	Major findings	
Meta-analyses								
Luan 2022	Meta-analysis of 5 studies (3 RCTs, 2 cohort studies)	7,732 adults T2DM	Dulaglutide, Exenatide, Liraglutide	Pre-treatment baseline	3 months-5 years	MMSE, MoCA	SMD = 0.33 95% CI = -0.03, 0.69 (p=0.017)	=
Norgaard 2022	Pooled analysis of 3 RCTs	15,820 adults T2DM	Liraglutide, Semaglutide	Placebo	1.3-3.8 years	Risk of any dementia	HR = 0.47 95% CI = 0.25, 0.86	+
Tang 2023	Meta-analysis of 4 studies (1 pooled analysis of 3 RCTs, 3 observational studies)	210,521 adults T2DM	Any GLP-1RAs	Nonusers of GLP-1RAs	3.6-7.4 years	Risk of any dementia	RR = 0.72 95% CI = 0.54, 0.97 (p=0.000)	+
Tian 2023	Network meta-analysis of 27 studies (4 for GLPl-RAs: 1 RCT, 3 case-control studies)	149,560 adults T2DM	Dulaglutide, Exenatide, Liraglutide	Nonusers of GLP-1RAs	4-7.2 years	Risk of any dementia	OR = 0.34 95% CI = 0.14, 0.85 (p=0.021)	+

***Legend:*** + : positive effect; = : no effect; - : negative effect. Values are mean±SD unless otherwise specified. Study ID reports the first author and year only.

GLP1-RA: Glucagon-Like Peptide-1 Receptor Agonist; HR: Hazard Ratio; MMSE: Mini-Mental State Examination; MoCA: Montreal Cognitive Assessment; OR: Odds Ratio; RCT: Randomised Controlled Trial; RR: Relative Risk; T2DM: Type 2 Diabetes Mellitus; SMD: Standardised Mean Difference.

**Table 2:** Clinical studies of GLP-1RAs for substance use disorders

Study ID	Design	Population	Intervention/ Exposure	Comparison	Follow up	Outcomes	Major Findings	
Alcohol								
Klausen 2022	RCT	127 adults AUD	Exenatide	Placebo	6 months	Number of heavy drinking days	Estimated treatment difference: 6.0 95% CI = -7.4, 19.4 (p=0.37)	=
Wang 2024a	Historical cohort	83,825 adults obesity	Semaglutide	Non-GLP1-RA anti-obesity medications	1 year	Incident AUD	HR = 0.50 95% CI = 0.39, 0.63	+
Wang 2024a	Historical cohort	589,803 adults T2DM	Semaglutide	Non-GLP1-RA anti-obesity medications	1 year	Incident AUD	HR = 0.61 95% CI = 0.50, 0.75	+
Wium- Andersen 2022a	Cohort	87,676 new users of GLP1-RAs or DPP-4 inhibitors	GLP1-RAs	DPP-4 inhibitors	4.1 years	Incident alcohol- related event	HR = 0.46 95% CI = 0.24, 0.86	+
Cocaine								
Angarita 2021	RCT	13 adults cocaine use disorder, non-treatment-seeking	Exenatide	Placebo	2 days	Behavioural and subjective effects of cocaine	“Acute pretreatment with exenatide vs placebo did not change cocaine infusions, self-reported euphoria, or wanting of cocaine.”	=
Yammine 2023	Case series	3 adults cocaine use disorder	Exenatide	-	6 weeks	Feasibility and safety	100% attendance and compliance. Positive end-of- study satisfaction ratings. Medication was well tolerated and without unexpected or severe adverse events.	+
Cannabis								
Wang 2024b	Historical cohort	85,223 adults obesity	Semaglutide	Non-GLP1-RA anti-obesity medications	1 year	Incident CUD	HR = 0.56 95% CI = 0.42, 0.75	+
Wang 2024b	Historical cohort	596,045 adults T2DM	Semaglutide	Non-GLP1-RA anti-obesity medications	1 year	Incident CUD	HR = 0.40 95% CI = 0.29, 0.56	+
Nicotine								
Lengsfeld 2023	RCT	255 adult smokers	Dulaglutide	Placebo	3 months	Point prevalence abstinence	Estimated difference in proportions: −1.9% 95% CI = -10.7, 14.4 (p=0.859)	+
Yammine 2021	RCT	84 adult smokers prediabetes or overweight	Exenatide (+NRT)	Placebo (+NRT)	6 weeks	7-day point Prevalence abstinence	RR = 1.7 95% credible interval = 0.96, 3.27 (PP=96.5%)	+

***Legend:*** + : positive effect; = : no effect; - : negative effect. Values are mean±SD unless otherwise specified. Study ID reports the first author and year only.

AUD: Alcohol Use Disorder; CUD: Cannabis Use Disorder; DPP-4: Dipeptidyl Peptidase-4; GLP1-RA: Glucagon-Like Peptide-1 Receptor Agonist; HR: Hazard Ratio; MAST: Michigan Alcohol Screening Tool; NRT: Nicotine Replacement Therapy; PP: Posterior Probability; RCT: Randomised Controlled Trial; T2DM: Type 2 Diabetes Mellitus.

**Table 3:** Clinical studies of GLP-1RAs for psychotic disorders, meta-analyses

Study ID	Design	Population	Intervention/ Exposure	Comparison	Follow-up	Outcomes	Major Findings	
Meta-analyses								
Khaity 2023	Meta-analysis (7 RCTs)	398 adults obesity on antipsychotics	Exenatide or Liraglutide	Placebo	3-6 months	BMI (kg/m^2^)	MD = -1.09 95% CI = -1.25, 0.93 (p<0.00001)	+
						Waist circumference (cm)	MD = -3.66 95% CI = -3.89, -3.44, (p<0.00001)	+
						Blood pressure (mmHg)	SBP: MD = -3.07 95% CI = -3.61, -2.53 (p<0.00001) DBP: MD = -2.02 95% CI = -2.42, -1.62 (p<0.00001)	+
Patoulias 2023	Meta-analysis (4 RCTs)	199 adults obesity on antipsychotics	Exenatide or Liraglutide	Placebo or Usual care	3-6 months	BMI (kg/m^2^)	MD = -1.04 95% CI = -1.92, -0.17 (p=0.02)	+
						Waist circumference (cm)	MD = -3.20 95% CI = -6.47, 0.08 (p=0.06)	=
						Blood pressure (mmHg)	SBP: MD = -1.44 95% CI = -5.38, 2.50 (p=0.47) DBP: MD = -1.35 95% CI = -5.62, 2.91 (p=0.53)	=
						Lipid profile	HDL: MD=0.09 95% CI = 0.01, 0.17 (p=0.03) LDL: MD = -0.31 95% CI = -0.46, 0.16 (p<0.0001)	+
Siskind 2019	Meta-analysis (3 RCTs)	168 adults obesity on antipsychotics	Exenatide or Liraglutide	Placebo or Usual care	3-6 months	BMI (kg/m^2^)	-1.19 ± 0.22(SE) (p<0.001)	+
						Waist circumference (cm)	-3.00 ± 0.68(SE) (p<0.001)	+
						Blood pressure (mmHg)	SBP: -1.89 ± 1.61(SE) (p=0.241) DBP: -1.91±1.17 (p=0.104)	=
						HbA1c	-3.25 ± 0.66(SE) (p<0.001)	+
						Lipid profile	No significant differences in HDL, LDL, TG	=
Wang 2021	Meta-analysis (4 RCTs)	219 adults obesity on atypical antipsychotics	Exenatide or Liraglutide	Placebo	3-6 months	BMI (kg/m^2^)	WMD = -1.0 95% CI = -1.8, -0.22	+
						Waist circumference (cm)	WMD = -2.29 95% CI = -4.63, -0.03	+
						Blood pressure (mmHg)	DBP: WMD = -2.98 95% CI = -6.06, -0.02 “SBP was not significantly changed after treatment.”	+

***Legend:*** + : positive effect; = : no effect; - : negative effect. Values are mean±SD unless otherwise specified. Study ID reports the first author and year only.

BMI: Body Mass Index; DBP: Diastolic Blood Pressure; GLP1-RA: Glucagon-Like Peptide-1 Receptor Agonist; HbA1c: Haemoglobin A1c; HDL: High Density Lipoprotein; LDL: Low Density Lipoprotein; MD: Mean Difference; RCT: Randomised Controlled Trial; SBP: Systolic Blood Pressure; SE: Standard Error; T2DM: Type 2 Diabetes Mellitus; TG: Triglycerides; WMD: Weighted Mean Difference.

**Table 4:** Clinical studies of GLP-1RAs for mood and anxiety disorders, effects in patients with mood disorders

Study ID	Design	Population	Intervention/Exposure	Comparison	Follow up	Outcomes	Major Findings	
Non-randomised studies								
Cuomo 2019	Historical cohort	29 adults BAD or MDD and obesity	Liraglutide	Pre-treatment baseline	6 months	Acceptability, adverse events	“No patient showed a worsening of the psychiatric condition due to liraglutide treatment [...] 48% completed the study”	=
Mansur 2017a	Nonrandomised open-label trial	19 adults BAD or MDD	Liraglutide	Pre-treatment baseline	4 weeks	Executive function (TMTB)	Cohen’s d = 0.64 (p=0.009)	+
Mansur 2017b	Nonrandomised open-label trial	19 adults BAD or MDD	Liraglutide	Pre-treatment baseline	4 weeks	Brain volumes (MRI)	“Increase in frontal and striatal volumes correlated BMI changes (r = -0.561, p=0.042 in left superior frontal area) [...] changes in brain volumes associated with improvement in executive function (r = 0.698, p=0.003 in right superior frontal area)”	+
Case series / reports								
Kohen 2008	Case report	1 older adult MDD and diabetes	Exenatide	NA	1-3 months	Relapse of Depressive symptoms	“Depressive symptoms resolved when off the medication and recurred when the patient was rechallenged with it”	-
Li 2023	Case series	1 adult without history of depression	Semaglutide	NA	1 month	Incidence of Depressive symptoms	“Occurrence of depressive symptoms, relieved by stopping Semaglutide”	-
						Relapse of Depressive symptoms	“Relapse of depressive symptoms relieved by stopping Semaglutide”	-

***Legend:*** + : positive effect; = : no effect; - : negative effect. Values are mean±SD unless otherwise specified. Study ID reports the first author and year only.

BAD: Bipolar Affective Disorder; BMI: Body Mass Index; GLP1-RA: Glucagon-Like Peptide-1 Receptor Agonist; MDD: Major Depressive Disorder; MRI: Magnetic Resonance Imaging; NA: Not Available; TMTB: Trail Making Test B

**Table 5:** Clinical studies of GLP-1RAs for mood and anxiety disorders, effects on depressive symptoms in patients with other comorbidities, meta-analyses

Study ID	Design	Population	Intervention/Exposure	Comparison	Follow-up	Outcomes	Major Findings	
Meta-analyses								
Chen 2024a	Meta-analysis of 6 studies (5 RCTs, 1 cohort study)	2,071 adults T2DM or Parkinson’s disease	Exenatide, Liraglutide	Placebo, Other antidiabetic	6 months- 1 year	Any depression rating scale	SMD = -0.12 95% CI = -0.21, - 0.03 (p<0.01)	+
O’Neil 2017	Pooled analysis of 5 RCTs	5,325 adults obesity	Liraglutide	Placebo	8 months- 3 years	PHQ-9	MD = -0.02 95% CI = -0.17, 0.12	=
						Self-reported suicidal ideation or behaviour	Liraglutide: 0.3%, placebo: 0.1%	-
Pozzi 2019	Meta-analysis of studies (1 pooled analysis of 5 RCTs, 3 clinical trials, 1 open-label extension study, 3 observational studies)	6,914 adults overweight/obesity and T2DM	Exenatide, Liraglutide	Placebo, Other antidiabetic	6 months- 1 year	Any depression rating scale	χ^2^ = 1.14, df = 1 (p=0.29)	=

***Legend:*** + : positive effect; = : no effect; - : negative effect. Values are mean±SD unless otherwise specified. Study ID reports the first author and year only.

GLP1-RA: Glucagon-Like Peptide-1 Receptor Agonist; MD: Mean Difference; PHQ-9: Patient Health Questionnaire; RCT: Randomised Controlled Trial; SMD: Standardised Mean Difference; T2DM: Type 2 Diabetes Mellitus.

**Table 6:** Clinical studies of GLP-1RAs for eating disorders

Study ID	Design	Population	Intervention/Exposure	Comparison	Follow-up	Outcomes	Major Findings	
Binge eating disorder								
Allison 2023	Pilot RCT	27 adults BED	Liraglutide	Placebo	4 months	OBEs /week	Liraglutide: -4.0 ± 0.6(SE), Placebo: -2.5 ± 0.5(SE) MD = 1.2 95% CI = 1.3, 2.0 (p=0.37)	=
DaPorto 2020	Pilot RCT (open-label)	60 adults BED and T2DM on Metformin	Dulaglutide	Gliclazide	3 months	BES score	Liraglutide: -12.067, Gliclazide: -0.467 (p<0.0001)	+
Richards 2023	Historical cohort	48 adults BED (moderate to severe)	Semaglutide	Other antiobesity medication (OAOM)	6 months	BES score	Semaglutide only: 14±8.2 (range -2.0 to 25.0) Semaglutide + OAOM: 12.9±8.9 (range 0 to 29.0) OAOM: 5.9±9.1 (range -7.0 to 24.0) (Semaglutide ± OAOM vs OAOM p<0.01)	+
Robert 2015	Pilot RCT	44 adults obesity and sub-clinical binge eating	Liraglutide + diet + exercise	Diet + exercise only	3 months	BES score	Liraglutide baseline: 20 (IQR 18.0 - 27.0), after treatment: 11 (IQR 7.0 - 16.0) (p<0.001) Control baseline: 22 (IQR 20.0 − 28.0), after treatment: 18 (IQR 12.0 - 22.0) (p<0.001)	+
Eating disorder psychopathology in co-morbid conditions								
Chao 2019	Exploratory RCT	150 adults obesity	IBT + Liraglutide or Multicompone nt (diet + IBT + Liraglutide)	IBT only	1 year	EDE-Q	Liraglutide + IBT: -0.6±0.1 (p<0.001) Multicomponent: -0.8±0.1 (p<0.001) IBT only: -0.4±0.1 (p<0.05) No significant differences between groups.	=
Jensterle 2014	Single-arm trial (openlabel)	36 adult women obesity and PCOS	Liraglutide (switched from metformin)	Pre-treatment baseline	3 months	TFEQ-R18	UE score baseline: 36.8±24.5, after treatment: 19.6±18.4 (p<0.001) EE score baseline: 49.9±33.3, after treatment: 28.5±26.9 (p<0.001)	+
Nicolau 2022	Prospective observational	69 adults obesity	Semaglutide	Pre-treatment baseline	3 months	Proportion of patients with EE (EE-Q)	Baseline: 72.5%, after treatment: 11.5% (p<0.001) “Amelioration of EE at 3 months of treatment with Semaglutide was associated with a greater weight loss (p=0.0003).”	+

***Legend***: + : positive effect; = : no effect; - : negative effect. Values are mean±SD unless otherwise specified. Study ID reports the first author and year only.

BED: Binge Eating Disorder; BES: Binge Eating Scale; EDE-Q: Eating Disorder Examination Questionnaire; EE: Emotional Eating; EE-Q: Emotional Eating Questionnaire; GLP1-RA: Glucagon-Like Peptide-1 Receptor Agonist; IBT: Intensive Behavioural Therapy; IQR: Interquartile Range; MD: Mean Difference; OBE: Objective Binge Episode; PCOS: Polycystic Ovary Syndrome; RCT: Randomised Controlled Trial; TFEQ-R18: Three- Factor Eating Questionnaire; T2DM: Type 2 Diabetes Mellitus; UE: Uncontrolled Eating.

## Data Availability

All data used for this manuscript are publicly available and are provided in the main text and supplementary information.

## Code Availability Statement

No code was used for this study.
