# Supplementary material for: An analysis on the role of glucagon-like peptide 1 receptor agonists in cognitive and mental health disorders
Source: Nat Ment Health. Author manuscript; Available in PMC 2026 Jun 2. (PMC7619120; doi:10.1038/s44220-025-00390-x)
Supplement: Supplementary Information [file EMS209664-supplement-Supplementary_Information.pdf]

S1. Search methods

Database:
Medline (Ovid MEDLINE® Epub Ahead of Print, In-Process & Other Non-Indexed Citations, Ovid MEDLINE® Daily and Ovid MEDLINE®) 1946 to present

#	**Query**	**Results from 20 Nov 2023**
1	Glucagon-Like Peptide 1/ag, aa, de, pd, tu [Agonists, Analogs & Derivatives, Drug Effects, Pharmacology, Therapeutic Use]	3,091
2	Glucagon-Like Peptide-1 Receptor/ag, de, tu [Agonists, Drug Effects, Therapeutic Use]	2,145
3	glp-1 agonist.mp.	382
4	glp-1 analogue.mp.	648
5	glp-1 receptor agonist.mp.	1,583
6	glucagon-like peptide-1 receptor agonist.mp.	1,575
7	Liraglutide/aa, pd, tu [Analogs & Derivatives, Pharmacology, Therapeutic Use]	1,451
8	Exenatide/ag, aa, pd, tu [Agonists, Analogs & Derivatives, Pharmacology, Therapeutic Use]	485
9	Semaglutide.mp.	1,382
10	Dulaglutide.mp.	757
11	Tirzepatide.mp.	339
12	Albiglutide.mp.	244
13	Lixisenatide.mp.	595
14	Taspoglutide.mp.	63
15	1 or 2 or 3 or 4 or 5 or 6 or 7 or 8 or 9 or 10 or 11 or 12 or 13 or 14	9,602

Database:
Embase 1974 to present

#	Query	Results from 20 Nov 2023
1	glucagon like peptide 1/ct, cb, cm, dv, dt, pd [Clinical Trial, Drug Combination, Drug Comparison, Drug Development, Drug Therapy, Pharmacology]	2,175
2	glucagon like peptide 1 receptor/ct, cb, cm, dt, ec, pd [Clinical Trial, Drug Combination, Drug Comparison, Drug Therapy, Endogenous Compound, Pharmacology]	2,841
3	glucagon like peptide 1 receptor agonist/ct, cb, cm, dt, ec, pd [Clinical Trial, Drug Combination, Drug Comparison, Drug Therapy, Endogenous Compound, Pharmacology]	6,257
4	1 or 2 or 3	10,618

Database:
Cochrane CENTRAL
Date Run: 21/11/2023 08:49:14

ID Search Hits

#1 MeSH descriptor: [Glucagon-Like Peptide 1] explode all trees 2196

#2 MeSH descriptor: [Glucagon-Like Peptide-1 Receptor] explode all trees 326

#3 #1 OR #2 2375

2367 Trials matching "#3 - #1 OR #2"

Database:
PsycINFO 1806 to present

#	Query	Results from 20 Nov 2023
1	glp-1.mp.	626
2	glp-1 agonist.mp.	30
3	glp-1 receptor.mp.	208

4	Dulaglutide.mp.	9
5	Albiglutide.mp.	1
6	Liraglutide.mp.	127
7	Semaglutide.mp.	14
8	Exenatide.mp.	77
9	Taspoglutide.mp.	0
10	Lixisenatide.mp.	9
11	Tirzepatide.mp.	1
12	glucagon-like peptide 1.mp.	728
13	glp-1 analogue.mp.	34
14	glucagon-like peptide.mp.	755
15	1 or 2 or 3 or 4 or 5 or 6 or 7 or 8 or 9 or 10 or 11 or 12 or 13 or 14	884

S2. Miscellaneous clinical studies

Supplementary Table S2. Summary of included studies regarding the adverse psychiatric outcomes associated with GLP1-RAs

Study ID	Study design	Population	Exposure	Comparison	Follow-up	Outcomes	Major Findings
Chen 2023	Pharmacovigilance study	507,514 AE reports	Albiglutide, Dulaglutide, Exenatide, Liraglutide, Lixisenatide, Semaglutide	Any drug in database	18 years	Incidence of suicidal and self-injurious behaviour	534 reports, including suicidal ideation (n = 275, 51.50%)<br><br>Disproportionality analysis: No over-reporting of suicide/ self-injury with GLP1-RA (ROR = 0.16, 95%CI = 0.15, 0.18 p<0.001)<br><br>No over-reporting of suicide/ self-injury with GLP1-RA before or after the COVID-19 pandemic outbreak.
Chen 2024b	Pharmacovigilance study	181,238 AE reports	Dulaglutide, Exenatide, Liraglutide, Lixisenatide, Semaglutide or Tirzepatide)	-	19 years	Incidence of any psychiatric AEs	8,240 reports, including insomnia (n = 1,198, 11.7%), anxiety (n = 1,131, 11.04%), nervousness (n = 941, 9.19%), depression (n = 770, 7.52%).
						Median time to onset of psychiatric AEs	31 days (IQR 7-145.4 days)
De Giorgi 2024	Historical cohort	130,352 patients	Semaglutide	Sitagliptin, Empagliflozin, Glipizide	1 year	Incidence of any neuropsychiatric diagnosis	"Semaglutide is not associated with higher 12-month risk of adverse neuropsychiatric outcomes compared to other antidiabetic medications"
Silverii 2024	Meta-analysis of 31 RCTs	84,713 patients	Any GLP1-RA	Placebo	>1 year	Incidence of any psychiatric AEs	OR = 0.97<br>95% CI = 0.83, 1.15 (p=0.76)
Tobaiqy 2024	Pharmacovigilance study	31,444 AE reports	Liraglutide, Semaglutide or Tirzepatide	-	1.5 years	Incidence of any psychiatric AEs	372 reports (1.18%), including depression (n = 187, 50.3%), anxiety (n = 144, 38.7%), suicidal ideation (n = 73, 19.6%)
Zhou 2024	Pharmacovigilance study	8,857,300 cases	Albiglutide, Dulaglutide, Exenatide, Liraglutide, Semaglutide	-	5 years	Incidence of suicidal and self-injurious behaviour	204 reports<br>Disproportionality analysis did not indicate an association between GLP-1RAs and suicide / self-injury

Legend: Values are mean±SD unless otherwise specified. Study ID reports the first author and year only.
AE: Adverse Events; GLP1-RA: Glucagon-Like Peptide 1 Receptor Agonist; OR: Odds Ratio; RCT: Randomised Controlled Trial, ROR: Reporting Odds Ratio.

S3. Ongoing/planned trials

Supplementary Table S3. Summary of ongoing/planned clinical studies of GLP1-RAs in psychiatric populations or related to mental health outcomes.

Study ID	Study design	Population	Exposure/Intervention	Comparison	Follow-up	Outcomes	Stage
Cognitive disorders							
GLP1-RAs effect on dementia risk / cognitive outcomes in patients with diabetes							
Davidy 2023	RCT (protocol)	80 older adults metabolic syndrome and MCI	Dulaglutide + Intranasal insulin	Placebo	1 year	Feasibility study study (adherence, safety)	Ongoing
NCT05313529	RCT	324 patients T2DM and MCI	Liraglutide	Other oral antidiabetics: Empagliflozin, Linagliptin	1 year	Change in cognitive function (MoCA)	To be completed 2027
NCT05360147	RCT	30 patients T2DM	Liraglutide	Other oral antidiabetics, insulin	3 months	Change in cognitive function (MMSE)	Completed 2021, unpublished
NCT02847403	RCT	40 patients dysglycaemia /prediabetes and MCI	Exenatide	Placebo	8 months	ADAS-cog score	Completed 2019, unpublished
GLP1-RAs in patients with cognitive impairment (without physical comorbidities)							
Femminella 2019	RCT (protocol)	204 patients MCI or probable AD, not on any antidiabetics	Liraglutide	Placebo	1 year	Change in cerebral glucose metabolic rate	Completed 2019, only preliminary results published (Edison, 2021)
NCT04777396	RCT	1840 patients MCI or mild AD	Semaglutide	Placebo	>3 years	CDR-SB score	To be completed 2026
NCT04777409	RCT	1840 patients MCI or mild AD	Semaglutide	Placebo	>3 years	CDR-SB score	To be completed 2026
GLP1-RAs effects on cognition in healthy subjects							
NCT01550653	RCT	40 healthy volunteers	Liraglutide	Placebo	5 weeks	Change in performance in cognitive battery	Completed 2013, No published results found
Substance use disorders							
Alcohol							
NCT06015893	RCT (Phase 2)	52 patients AUD	Semaglutide	Placebo	6 months	Alcohol consumption (number of drinks consumed per week)	Estimated completion 2030
NCT05891587	RCT (Phase 2)	80 patients AUD	Semaglutide	Placebo	3 months	Alcohol consumption (number of drinks consumed per week)	Estimated completion 2025
NCT05520775	RCT (Phase 2)	48 patients AUD	Semaglutide	Placebo	2 months	Volume of alcohol consumption (in self-administration procedure), Breath alcohol concentration	Estimated completion April 2024

NCT05892432	RCT (Phase 2)	135 patients AUD	Semaglutide	Placebo	2 months	Cue craving visual analog score	Estimated completion 2025
NCT05895643	RCT (Phase 2)	108 patients AUD	Semaglutide	Placebo	6 months	Number of heavy drinking days	Estimated completion 2027
NCT03645408	RCT Crossover (Phase 1)	3 patients classified as "heavy drinkers"	Exenatide	Placebo	2 hours - after acute dose	Alcohol consumption (standard drink units)	Full report not published, preliminary results on clinicaltrials.gov: Exenatide: 0.93±0.87, Placebo: 2.78±2.66
Opiates							
NCT04199728	RCT	27 treatment-seeking adults opiate use disorder	Liraglutide	Placebo	1 month	Change in ambient drug craving and in self-reported cue-elicited drug craving as measured by VAS	Completed 2023 - results not yet published
Nicotine							
NCT03712098	RCT	40 adult smokers overweight BMI	Liraglutide	Placebo	8 months	7-day point prevalence abstinence rate (at 12 weeks)	Study completed 2022 – full results not yet published
NCT02690987	RCT crossover (Phase 1)	95 adults previous alcohol or tobacco dependence, overweight BMI	Exenatide	Placebo	4 years	fMRI measures during cigarette, alcohol and food picture evaluation task	Estimated completion 2020 - full results not yet published
Psychotic disorders							
NCT05333003	RCT	92 patients psychotic illness, on antipsychotic, BMI ≥30	Semaglutide	Placebo	8 months	Body weight	Estimated completion 2025
NCT05897398	Cohort	1000 patients severe eating disorder or iatrogenic obesity induced by psychotropics	Semaglutide	Baseline	1 year	Body weight	Estimated completion 2026
Sailer, 2019	RCT (protocol)	154 adults on a second-generation antipsychotic (Clozapine, Olanzapine)	Dulaglutide	Placebo	6 months (+1 year follow-up)	Body weight	Ongoing study
Sass, 2023	RCT (protocol)	104 patients schizophrenia and prediabetes or T2DM, on Clozapine / Olanzapine	Semaglutide	Placebo	6 months	HbA1c	Estimated completion 2024
Siskind, 2023	RCT (protocol)	80 patients schizophrenia and BMI ≥26 on Clozapine	Semaglutide	Placebo	9 months	Body weight	Ongoing study
Mood and anxiety disorders							

NCT04466345	RCT	60 patients MDD and pre-treatment cognitive dysfunction	Semaglutide	Placebo	4 months	Executive function	Estimated completion 2024
NCT05492305	Prospective cohort	20 patients on any GLP1-RA for obesity or diabetes	Any GLP1-RA	Baseline	3-4 months	Subjective effect on mental health (qualitative analysis, semi-structured interview)	Estimated completion 2023, full results not published

Legend: Values are mean±SD unless otherwise specified. Study ID reports the first author and year only.
AD: Alzheimer's Disease; ADAS: Alzheimer's Disease Assessment Scale; AUD: Alcohol Use Disorder; BED: Binge Eating Disorder; CDR-SB: Clinical Dementia Rating – Sum of Boxes; DSST: Digit Symbol Substitution Test; fMRI: functional Magnetic Resonance Imaging; GLP1-RA: Glucagon-Like Peptide 1 Receptor Agonist; HbA1c: Haemoglobin A1c; MCI: Mild Cognitive Impairment; MDD: Major Depressive Disorder; MMSE: Mini Mental State Examination; MoCA: Montreal Cognitive Assessment; RCT: Randomised Controlled Trial; T2DM: Type 2 Diabetes Mellitus; VAS: Visual Analog Score.

S4. List of pre-clinical/mechanistic studies on cognitive disorders

Abd El-Rady, N. M., Ahmed, A., Abdel-Rady, M. M. & Ismail, O. I. Glucagon-like peptide-1 analog improves neuronal and behavioral impairment and promotes neuroprotection in a rat model of aluminum-induced dementia. Physiological reports 8, e14651 (2021). https://doi.org:https://dx.doi.org/10.14814/phy2.14651

Abdelwahed, O. M., Tork, O. M., Gamal el Din, M. M., Rashed, L. & Zickri, M. Effect of glucagon-like peptide-1 analogue; Exendin-4, on cognitive functions in type 2 diabetes mellitus; possible modulation of brain derived neurotrophic factor and brain Visfatin. Brain Research Bulletin 139, 67-80 (2018). https://doi.org:https://dx.doi.org/10.1016/j.brainresbull.2018.02.002

Ahn, Y.-J. et al. Exendin-4 Pretreatment Attenuates Kainic Acid-Induced Hippocampal Neuronal Death. Cells 10 (2021). https://doi.org:https://dx.doi.org/10.3390/cells10102527

An, J. et al. Exenatide alleviates mitochondrial dysfunction and cognitive impairment in the 5xFAD mouse model of Alzheimer's disease. Behavioural Brain Research 370 (2019). https://doi.org:https://dx.doi.org/10.1016/j.bbr.2019.111932

An, J.-R. et al. Liraglutide Alleviates Cognitive Deficit in db/db Mice: Involvement in Oxidative Stress, Iron Overload, and Ferroptosis. Neurochemical research 47, 279-294 (2022). https://doi.org:https://dx.doi.org/10.1007/s11064-021-03442-7

Babic Perhoc, A. et al. Cognitive, behavioral and metabolic effects of oral galactose treatment in the transgenic Tg2576 mice. Neuropharmacology 148, 50-67 (2019). https://doi.org:https://dx.doi.org/10.1016/j.neuropharm.2018.12.018

Bae, C. S. & Song, J. The role of glucagon-like peptide 1 (GLP1) in type 3 diabetes: GLP-1 controls insulin resistance, neuroinflammation and neurogenesis in the brain. International Journal of Molecular Sciences 18, 2493 (2017). https://doi.org:https://dx.doi.org/10.3390/ijms18112493

Bailey, J. et al. GLP-1 receptor nitration contributes to loss of brain pericyte function in a mouse model of diabetes. Diabetologia 65, 1541-1554 (2022). https://doi.org:https://dx.doi.org/10.1007/s00125-022-05730-5

Bao, Y. et al. The neuroprotective effect of liraglutide is mediated by glucagon-like peptide 1 receptor-mediated activation of cAMP/PKA/CREB pathway. Cellular Physiology and Biochemistry 36, 2366-2378 (2015). https://doi.org:https://dx.doi.org/10.1159/000430199

Barilar, J. O., Knezovic, A., Homolak, J., Perhoc, A. B. & Salkovic-petrisic, M. Divergent Effect of Central Incretin Receptors Inhibition in a Rat Model of Sporadic Alzheimer's Disease. International Journal of Molecular Sciences 23, 548 (2022). https://doi.org:https://dx.doi.org/10.3390/ijms23010548

Batista, A. F. et al. The diabetes drug liraglutide reverses cognitive impairment in mice and attenuates insulin receptor and synaptic pathology in a non-human primate model of Alzheimer's disease. Journal of Pathology 245, 85-100 (2018). https://doi.org:https://dx.doi.org/10.1002/path.5056

Bomba, M. et al. Exenatide exerts cognitive effects by modulating the BDNF-TrkB neurotrophic axis in adult mice. Neurobiology of Aging 64, 33-43 (2018). https://doi.org:https://dx.doi.org/10.1016/j.neurobiolaging.2017.12.009

Bomba, M. et al. Exenatide reverts the high-fat-diet-induced impairment of BDNF signaling and inflammatory response in an animal model of Alzheimer's disease. Journal of Alzheimer's Disease 70, 793-810 (2019). https://doi.org:https://dx.doi.org/10.3233/JAD-190237

Cai, H.-Y. et al. Lixisenatide attenuates the detrimental effects of amyloid beta protein on spatial working memory and hippocampal neurons in rats. Behavioural Brain Research 318, 28-35 (2017). https://doi.org:https://dx.doi.org/10.1016/j.bbr.2016.10.033

Cai, H.-Y. et al. A GLP-1/GIP dual receptor agonist DA4-JC effectively attenuates cognitive impairment and pathology in the APP/PS1/tau model of Alzheimer's Disease. Journal of Alzheimer's Disease 83, 799-818 (2021). https://doi.org:https://dx.doi.org/10.3233/JAD-210256

Cai, X. S. et al. Glucagon-like peptide-1 (GLP-1) treatment ameliorates cognitive impairment by attenuating Arc expression in type 2 diabetic rats. Medical Science Monitor 23, 4334-4342 (2017). https://doi.org:https://dx.doi.org/10.12659/MSM.903252

Candeias, E. et al. Brain GLP-1/IGF-1 Signaling and Autophagy Mediate Exendin-4 Protection Against Apoptosis in Type 2 Diabetic Rats. Molecular Neurobiology 55, 4030-4050 (2018). https://doi.org:https://dx.doi.org/10.1007/s12035-017-0622-3

Cao, L. et al. A novel dual GLP-1 and GIP incretin receptor agonist is neuroprotective in a mouse model of Parkinson's disease by reducing chronic inflammation in the brain. NeuroReport: For Rapid Communication of Neuroscience Research 27, 384-391 (2016). https://doi.org:https://dx.doi.org/10.1097/WNR.0000000000000548

Cao, Y. et al. DA5-CH, a novel GLP-1/GIP dual agonist, effectively ameliorates the cognitive impairments and pathology in the APP/PS1 mouse model of Alzheimer's disease. European Journal of Pharmacology 827, 215-226 (2018). https://doi.org:https://dx.doi.org/10.1016/j.ejphar.2018.03.024

Carranza-Naval, M. J. et al. Liraglutide reduces vascular damage, neuronal loss, and cognitive impairment in a mixed murine model of Alzheimer's disease and type 2 diabetes. Frontiers in Aging Neuroscience 13 (2021). https://doi.org:https://dx.doi.org/10.3389/fnagi.2021.741923

Chalichem, N. S. S., Sai Kiran, P. S. S. & Basavan, D. Possible role of DPP4 inhibitors to promote hippocampal neurogenesis in Alzheimer's disease. Journal of Drug Targeting 26, 670-675 (2018). https://doi.org:https://dx.doi.org/10.1080/1061186X.2018.1433682

Chang, Y.-F., Zhang, D., Hu, W.-M., Liu, D.-X. & Li, L. Semaglutide-mediated protection against Abeta correlated with enhancement of autophagy and inhibition of apotosis. Journal of clinical neuroscience : official journal of the Neurosurgical Society of Australasia 81, 234-239 (2020). https://doi.org:https://dx.doi.org/10.1016/j.jocn.2020.09.054

Chen, S. et al. Evidence of metabolic memory-induced neurodegeneration and the therapeutic effects of glucagon-like peptide-1 receptor agonists via Forkhead box class O. Biochimica et biophysica acta. Molecular basis of disease 1865, 371-377 (2019). https://doi.org:https://dx.doi.org/10.1016/j.bbadis.2018.11.012

Chen, S. et al. DPP-4 inhibitor improves learning and memory deficits and AD-like neurodegeneration by modulating the GLP-1 signaling. Neuropharmacology 157 (2019). https://doi.org:https://dx.doi.org/10.1016/j.neuropharm.2019.107668

Chen, X. et al. Effect of semaglutide and empagliflozin on cognitive function and hippocampal phosphoproteomic in obese mice. Frontiers in pharmacology 14, 975830 (2023). https://doi.org:https://dx.doi.org/10.3389/fphar.2023.975830

Chen, Y. et al. Regulatory mechanisms of the green alga Ulva lactuca oligosaccharide via the metabolomics and gut microbiome in diabetic mice. Current Research in Food Science 5, 1127-1139 (2022). https://doi.org:https://dx.doi.org/10.1016/j.crfs.2022.07.003

Cisternas, P. & Inestrosa, N. C. Brain glucose metabolism: Role of Wnt signaling in the metabolic impairment in Alzheimer's disease. Neuroscience and Biobehavioral Reviews 80, 316-328 (2017). https://doi.org:https://dx.doi.org/10.1016/j.neubiorev.2017.06.004

Csajbok, E. A. et al. Expression of GLP-1 receptors in insulin-containing interneurons of rat cerebral cortex. Diabetologia 62, 717-725 (2019). https://doi.org:https://dx.doi.org/10.1007/s00125-018-4803-z

Cui, S.-S., Feng, X.-B., Zhang, B.-H., Xia, Z.-Y. & Zhan, L.-Y. Exendin-4 attenuates pain-induced cognitive impairment by alleviating hippocampal neuroinflammation in a rat model of spinal nerve ligation. Neural regeneration research 15, 1333-1339 (2020). https://doi.org:https://dx.doi.org/10.4103/1673-5374.272620

Day, S. M. et al. Glucagon-Like Peptide-1 Cleavage Product Improves Cognitive Function in a Mouse Model of Down Syndrome. eNeuro 6 (2019). https://doi.org:https://dx.doi.org/10.1523/ENEURO.0031-19.2019

Denver, P., Gault, V. A. & McClean, P. L. Sustained high-fat diet modulates inflammation, insulin signalling and cognition in mice and a modified xenin peptide ameliorates neuropathology in a chronic high-fat model. Diabetes, Obesity and Metabolism 20, 1166-1175 (2018). https://doi.org:https://dx.doi.org/10.1111/dom.13210

Diz-Chaves, Y., Herrera-Perez, S., Gonzalez-Matias, L. C. & Mallo, F. Effects of Glucagon-like peptide 1 (GLP-1) analogs in the hippocampus. Vitamins and hormones 118, 457-478 (2022). https://doi.org:https://dx.doi.org/10.1016/bs.vh.2021.12.005

Dong, Q. et al. Sitagliptin protects the cognition function of the Alzheimer's disease mice through activating glucagon-like peptide-1 and BDNF-TrkB signalings. Neuroscience letters 696, 184-190 (2019). https://doi.org:https://dx.doi.org/10.1016/j.neulet.2018.12.041

Duarte, A. I. et al. Liraglutide Protects Against Brain Amyloid-beta1-42 Accumulation in Female Mice with Early Alzheimer's Disease-Like Pathology by Partially Rescuing Oxidative/Nitrosative Stress and Inflammation. International journal of molecular sciences 21 (2020). https://doi.org:https://dx.doi.org/10.3390/ijms21051746

Filchenko, I., Simanenkova, A., Chefu, S., Kolpakova, M. & Vlasov, T. Neuroprotective effect of glucagon-like peptide-1 receptor agonist is independent of glycaemia normalization in type two diabetic rats. Diabetes & vascular disease research 15, 567-570 (2018). https://doi.org:https://dx.doi.org/10.1177/1479164118788079

Garabadu, D. & Verma, J. Exendin-4 attenuates brain mitochondrial toxicity through PI3K/Akt-dependent pathway in amyloid beta (1-42)-induced cognitive deficit rats. Neurochemistry international 128, 39-49 (2019). https://doi.org:https://dx.doi.org/10.1016/j.neuint.2019.04.006

Gault, V. A. & Holscher, C. GLP-1 agonists facilitate hippocampal LTP and reverse the impairment of LTP induced by beta-amyloid. European journal of pharmacology 587, 112-117 (2008). https://doi.org:https://dx.doi.org/10.1016/j.ejphar.2008.03.025

Gault, V. A., Porter, W. D., Flatt, P. R. & Holscher, C. Actions of exendin-4 therapy on cognitive function and hippocampal synaptic plasticity in mice fed a high-fat diet. International Journal of Obesity 34, 1341-1344 (2010). https://doi.org:https://dx.doi.org/10.1038/ijo.2010.59

Gejl, M. et al. Blood-Brain Glucose Transfer in Alzheimer's disease: effect of GLP-1 Analog Treatment. 7, 17490 (2017). https://doi.org:10.1038/s41598-017-17718-y

Gejl, M. et al. In Alzheimer's disease, 6-month treatment with GLP-1 analog prevents decline of brain glucose metabolism: Randomized, placebo-controlled, double-blind clinical trial. Frontiers in Aging Neuroscience 8 (2016).

Gharagozloo, M. et al. The Effects of NLY01, a Novel Glucagon-Like Peptide-1 Receptor Agonist, on Cuprizone-Induced Demyelination and Remyelination: Challenges and Future Perspectives. Neurotherapeutics : the journal of the American Society for Experimental NeuroTherapeutics 20, 1229-1240 (2023). https://doi.org:https://dx.doi.org/10.1007/s13311-023-01390-4

Ghosh, P. et al. Targeting redox imbalance in neurodegeneration: characterizing the role of GLP-1 receptor agonists. Theranostics 13, 4872-4884 (2023). https://doi.org:https://dx.doi.org/10.7150/thno.86831

Greig, N. H. et al. New therapeutic strategies and drug candidates for neurodegenerative diseases: p53 and TNF-alpha inhibitors, and GLP-1 receptor agonists. Annals of the New York Academy of Sciences 1035, 290-315 (2004). https://doi.org:https://dx.doi.org/10.1196/annals.1332.018

Guan, T. et al. Dulaglutide Improves Gliosis and Suppresses Apoptosis/Autophagy Through the PI3K/Akt/mTOR Signaling Pathway in Vascular Dementia Rats. Neurochemical research 48, 1561-1579 (2023). https://doi.org:https://dx.doi.org/10.1007/s11064-022-03853-0

Gumuslu, E. et al. Exenatide upregulates gene expression of glucagon-like peptide-1 receptor and nerve growth factor in streptozotocin/nicotinamide-induced diabetic mice. Fundamental and Clinical Pharmacology 32, 174-180 (2018). https://doi.org:https://dx.doi.org/10.1111/fcp.12329

Guo, X. et al. Tirzepatide ameliorates spatial learning and memory impairment through modulation of aberrant insulin resistance and inflammation response in diabetic rats. Frontiers in pharmacology 14, 1146960 (2023). https://doi.org:https://dx.doi.org/10.3389/fphar.2023.1146960

Han, W., Xu, X., Shi, J., Li, X. & Cai, H. Prediction and validation of potential targets for the glucagon-like peptide-1 receptor agonist in the treatment of Alzheimer's disease. Chinese Journal of Tissue Engineering Research 28, 2568-2573 (2024). https://doi.org:https://dx.doi.org/10.12307/2024.306

Han, W.-N. et al. Liraglutide protects against amyloid-beta protein-induced impairment of spatial learning and memory in rats. Neurobiology of Aging 34, 576-588 (2013). https://doi.org:https://dx.doi.org/10.1016/j.neurobiolaging.2012.04.009

Hansen, H. H. et al. The GLP-1 receptor agonist liraglutide reduces pathology-specific tau phosphorylation and improves motor function in a transgenic hTauP301L mouse model of tauopathy. Brain Research 1634, 158-170 (2016). https://doi.org:https://dx.doi.org/10.1016/j.brainres.2015.12.052

Hansen, H. H. et al. The GLP-1 receptor agonist liraglutide improves memory function and increases hippocampal CA1 neuronal numbers in a senescence-accelerated mouse model of Alzheimer's disease. Journal of Alzheimer's Disease 46, 877-888 (2015). https://doi.org:https://dx.doi.org/10.3233/JAD-143090

Hinkle, J. T., Dawson, V. L. & Dawson, T. M. The A1 astrocyte paradigm: New avenues for pharmacological intervention in neurodegeneration. Movement disorders : official journal of the Movement Disorder Society 34, 959-969 (2019). https://doi.org:https://dx.doi.org/10.1002/mds.27718

Hlscher, C. Potential role of glucagon-like peptide-1 (GLP-1) in neuroprotection. CNS Drugs 26, 871-882 (2012). https://doi.org:https://dx.doi.org/10.2165/11635890-000000000-00000

Holmes, S. M. The effect of glucagon-like peptide-1 (GLP-1) receptor agonists on cell viability, adam10 maturation and the proteolysis of adam10 substrates in Sh-Sy5y cells. Dissertation Abstracts International: Section B: The Sciences and Engineering 82, No-Specified (2021).

Holscher, C. Incretin analogues that have been developed to treat type 2 diabetes hold promise as a novel treatment strategy for Alzheimer's disease. Recent Patents on CNS Drug Discovery 5, 109-117 (2010). https://doi.org:https://dx.doi.org/10.2174/157488910791213130

Holscher, C. The incretin hormones glucagonlike peptide 1 and glucose-dependent insulinotropic polypeptide are neuroprotective in mouse models of Alzheimer's disease. Alzheimer's & Dementia: The Journal of the Alzheimer's Association 10, S47-S54 (2014). https://doi.org:https://dx.doi.org/10.1016/j.jalz.2013.12.009

Holscher, C. "The incretin hormones glucagonlike peptide 1 and glucose-dependent insulinotropic polypeptide are neuroprotective in mouse models of Alzheimer's disease": Corrigendum. Alzheimer's & Dementia: The Journal of the Alzheimer's Association 11, 1395 (2015). https://doi.org:https://dx.doi.org/10.1016/j.jalz.2015.10.003

Holscher, C. Novel dual GLP-1/GIP receptor agonists show neuroprotective effects in Alzheimer's and Parkinson's disease models. Neuropharmacology 136, 251-259 (2018). https://doi.org:https://dx.doi.org/10.1016/j.neuropharm.2018.01.040

Holscher, C. Two dual GLP-1/GIP receptor agonists show superior protective properties compared to liraglutide in the APP/PS1 mouse model of Alzheimer's disease. Alzheimer's and Dementia 17, e050388 (2021). https://doi.org:https://dx.doi.org/10.1002/alz.050388

Holscher, C. Glucagon-like peptide 1 and glucose-dependent insulinotropic peptide hormones and novel receptor agonists protect synapses in Alzheimer's and Parkinson's diseases. Frontiers in Synaptic Neuroscience 14, 955258 (2022). https://doi.org:https://dx.doi.org/10.3389/fnsyn.2022.955258

Holubova, M. et al. Liraglutide and a lipidized analog of prolactin-releasing peptide show neuroprotective effects in a mouse model of beta-amyloid pathology. Neuropharmacology 144, 377-387 (2019). https://doi.org:https://dx.doi.org/10.1016/j.neuropharm.2018.11.002

Hunter, K. & Holscher, C. Drugs developed to treat diabetes, liraglutide and lixisenatide, cross the blood brain barrier and enhance neurogenesis. BMC Neuroscience 13 (2012). https://doi.org:https://dx.doi.org/10.1186/1471-2202-13-33

Iwai, T., Ito, S., Tanimitsu, K., Udagawa, S. & Oka, J.-I. Glucagon-like peptide-1 inhibits LPS-induced IL-1beta production in cultured rat astrocytes. Neuroscience research 55, 352-360 (2006).

Iwai, T. et al. Glucagon-like peptide-1 protects synaptic and learning functions from neuroinflammation in rodents. Journal of Neuroscience Research 92, 446-454 (2014). https://doi.org:https://dx.doi.org/10.1002/jnr.23335

Iwasa, K. et al. A peripheral lipid sensor GPR120 remotely contributes to suppression of PGD2-microglia-provoked neuroinflammation and neurodegeneration in the mouse hippocampus. Journal of neuroinflammation 18, 304 (2021). https://doi.org:https://dx.doi.org/10.1186/s12974-021-02361-2

Jantrapirom, S. et al. Liraglutide Suppresses Tau Hyperphosphorylation, Amyloid Beta Accumulation through Regulating Neuronal Insulin Signaling and BACE-1 Activity. International journal of molecular sciences 21 (2020). https://doi.org:https://dx.doi.org/10.3390/ijms21051725

Ji, C. et al. A novel dual GLP-1 and GIP receptor agonist is neuroprotective in the MPTP mouse model of Parkinson's disease by increasing expression of BNDF. Brain Research 1634, 1-11 (2016). https://doi.org:https://dx.doi.org/10.1016/j.brainres.2015.09.035

Jia, X.-T. et al. Exendin-4, a glucagon-like peptide 1 receptor agonist, protects against amyloid-beta peptide-induced impairment of spatial learning and memory in rats. Physiology & Behavior 159, 72-79 (2016). https://doi.org:https://dx.doi.org/10.1016/j.physbeh.2016.03.016

Jia, Y., Gong, N., Li, T. F., Zhu, B. & Wang, Y. X. Peptidic exenatide and herbal catalpol mediate neuroprotection via the hippocampal GLP-1 receptor/beta-endorphin pathway. Pharmacological Research 102, 276-285 (2015). https://doi.org:https://dx.doi.org/10.1016/j.phrs.2015.10.008

Kamei, N. et al. Effective nose-to-brain delivery of exendin-4 via coadministration with cell-penetrating peptides for improving progressive cognitive dysfunction. Scientific reports 8, 17641 (2018). https://doi.org:https://dx.doi.org/10.1038/s41598-018-36210-9

Kang, X. et al. Exendin-4 ameliorates tau hyperphosphorylation and cognitive impairment in type 2 diabetes through acting on Wnt/beta-catenin/NeuroD1 pathway. Molecular medicine (Cambridge, Mass.) 29, 118 (2023). https://doi.org:https://dx.doi.org/10.1186/s10020-023-00718-2

Khalaf, M. M., El-Sayed, M. M., Kandeil, M. A. & Ahmed, S. A novel protective modality against rotenone-induced Parkinson's disease: A pre-clinical study with dulaglutide. International immunopharmacology 119, 110170 (2023). https://doi.org:https://dx.doi.org/10.1016/j.intimp.2023.110170

Khalilnezhad, A. & Taskiran, D. The investigation of protective effects of glucagon-like peptide-1 (GLP-1) analogue exenatide against glucose and fructose-induced neurotoxicity. International Journal of Neuroscience 129, 481-491 (2019). https://doi.org:https://dx.doi.org/10.1080/00207454.2018.1543671

Kimura, R. et al. Glucagon-like peptide-1 (GLP-1) protects against methylglyoxal-induced PC12 cell apoptosis through the PI3K/Akt/mTOR/GCLc/redox signaling pathway. Neuroscience 162, 1212-1219 (2009). https://doi.org:https://dx.doi.org/10.1016/j.neuroscience.2009.05.025

Knezovic, A. et al. Glucagon-like peptide-1 mediates effects of oral galactose in streptozotocin-induced rat model of sporadic Alzheimer's disease. Neuropharmacology 135, 48-62 (2018). https://doi.org:https://dx.doi.org/10.1016/j.neuropharm.2018.02.027

Kobayashi, K., Iwai, T., Sasaki-Hamada, S., Kamanaka, G. & Oka, J.-I. Exendin (5-39), an antagonist of GLP-1 receptor, modulates synaptic transmission via glutamate uptake in the dentate gyrus. Brain Research 1505, 1-10 (2013). https://doi.org:https://dx.doi.org/10.1016/j.brainres.2013.01.012

Kong, F.-J., Wu, J.-H., Sun, S.-Y., Ma, L.-L. & Zhou, J.-Q. Liraglutide ameliorates cognitive decline by promoting autophagy via the AMP-activated protein kinase/mammalian target of rapamycin pathway in a streptozotocin-induced mouse model of diabetes. Neuropharmacology 131, 316-325 (2018). https://doi.org:https://dx.doi.org/10.1016/j.neuropharm.2018.01.001

Kong, J., Wan, L., Wang, Y., Zhang, H. & Zhang, W. Liraglutide attenuates Abeta42 generation in APPswe/SH-SY5Y cells through the regulation of autophagy. Neuropsychiatric Disease and Treatment 16 (2020). https://doi.org:https://dx.doi.org/10.2147/NDT.S260160

Kong, X. et al. GLP-1 Receptor Agonist Inhibited the Activation of RIPK1 for Alleviation the Neuronal Death and Neuroinflammation in APP/PS1 Mice. International Journal of Peptide Research and Therapeutics 27, 1699-1707 (2021). https://doi.org:https://dx.doi.org/10.1007/s10989-021-10202-2

Larsson, M. et al. Diabetes negatively affects cortical and striatal GABAergic neurons: An effect that is partially counteracted by exendin-4. Bioscience Reports 36, e00421 (2016). https://doi.org:https://dx.doi.org/10.1042/BSR20160437

Lee, C. H. et al. Activation of Glucagon-Like Peptide-1 Receptor Promotes Neuroprotection in Experimental Autoimmune Encephalomyelitis by Reducing Neuroinflammatory Responses. Molecular Neurobiology 55, 3007-3020 (2018). https://doi.org:https://dx.doi.org/10.1007/s12035-017-0550-2

Lennox, R. et al. Comparison of the independent and combined effects of sub-chronic therapy with metformin and a stable GLP-1 receptor agonist on cognitive function, hippocampal synaptic plasticity and metabolic control in high-fat fed mice. Neuropharmacology 86, 22-30 (2014). https://doi.org:https://dx.doi.org/10.1016/j.neuropharm.2014.06.026

Li, C. et al. The novel GLP-1/GIP analogue DA5-CH reduces tau phosphorylation and normalizes theta rhythm in the icv. STZ rat model of AD. Brain and behavior 10, e01505 (2020). https://doi.org:https://dx.doi.org/10.1002/brb3.1505

Li, H. et al. GLP-1 receptor regulates cell growth through regulating IDE expression level in Abeta1-42-treated PC12 cells. Bioscience Reports 38, BSR20171284 (2018). https://doi.org:https://dx.doi.org/10.1042/BSR20171284

Li, L. The molecular mechanism of glucagon-like peptide-1 therapy in Alzheimer's disease, based on a mechanistic target of rapamycin pathway. CNS Drugs 31, 535-549 (2017). https://doi.org:https://dx.doi.org/10.1007/s40263-017-0431-2

Li, T. et al. A novel GLP-1/GIP/Gcg triagonist reduces cognitive deficits and pathology in the 3xTg mouse model of Alzheimer's disease. Hippocampus 28, 358-372 (2018). https://doi.org:https://dx.doi.org/10.1002/hipo.22837

Li, T. et al. A GLP-1/GIP/Gcg receptor triagonist improves memory behavior, as well as synaptic transmission, neuronal excitability and Ca2+ homeostasis in 3xTg-AD mice. Neuropharmacology 170 (2020). https://doi.org:https://dx.doi.org/10.1016/j.neuropharm.2020.108042

Li, Y. et al. Liraglutide is neurotrophic and neuroprotective in neuronal cultures and mitigates mild traumatic brain injury in mice. Journal of Neurochemistry 135, 1203-1217 (2015). https://doi.org:https://dx.doi.org/10.1111/jnc.13169

Li, Y. et al. GLP-1 receptor stimulation reduces amyloid-s peptide accumulation and cytotoxicity in cellular and animal models of Alzheimer's disease. Journal of Alzheimer's Disease 19, 1205-1219 (2010). https://doi.org:https://dx.doi.org/10.3233/JAD-2010-1314

Li, Y. et al. GLP-1 receptor stimulation reduces amyloid-beta peptide accumulation and cytotoxicity in cellular and animal models of Alzheimer's disease. Journal of Alzheimer's Disease 19, 1205-1219 (2010). https://doi.org:https://dx.doi.org/10.3233/JAD-2010-1314

Li, Y. et al. The metabolite GLP-1 (9-36) is neuroprotective and anti-inflammatory in cellular models of neurodegeneration. Journal of neurochemistry 159, 867-886 (2021). https://doi.org:https://dx.doi.org/10.1111/jnc.15521

Li, Y., Tweedie, D., Mattson, M. P., Holloway, H. W. & Greig, N. H. Enhancing the GLP-1 receptor signaling pathway leads to proliferation and neuroprotection in human neuroblastoma cells. Journal of Neurochemistry 113, 1621-1631 (2010). https://doi.org:https://dx.doi.org/10.1111/j.1471-4159.2010.06731.x

Li, Z. et al. Systemic GLP-1R agonist treatment reverses mouse glial and neurovascular cell transcriptomic aging signatures in a genome-wide manner. Communications biology 4, 656 (2021). https://doi.org:https://dx.doi.org/10.1038/s42003-021-02208-9

Lietzau, G., Nystrom, T., Ostenson, C. G., Darsalia, V. & Patrone, C. Type 2 diabetes-induced neuronal pathology in the piriform cortex of the rat is reversed by the GLP-1 receptor agonist Exendin-4. Oncotarget 7, 5865-5876 (2016). https://doi.org:https://dx.doi.org/10.18632/oncotarget.6823

Liu, J., Yin, F., Zheng, X., Jing, J. & Hu, Y. Geniposide, a novel agonist for GLP-1 receptor, prevents PC12 cells from oxidative damage via MAP kinase pathway. Neurochemistry international 51, 361-369 (2007).

Liu, J. et al. Neurotrophic property of geniposide for inducing the neuronal differentiation of PC12 cells. International journal of developmental neuroscience : the official journal of the International Society for Developmental Neuroscience 24, 419-424 (2006).

Liu, Y. et al. Multi-target PET evaluation in APP/PS1/tau mouse model of Alzheimer's disease. Neuroscience Letters 728, 134938 (2020). https://doi.org:https://dx.doi.org/10.1016/j.neulet.2020.134938

Long-Smith, C. M. et al. The diabetes drug liraglutide ameliorates aberrant insulin receptor localisation and signalling in parallel with decreasing both amyloid-beta plaque and glial pathology in a mouse model of Alzheimer's disease. NeuroMolecular Medicine 15, 102-114 (2013). https://doi.org:https://dx.doi.org/10.1007/s12017-012-8199-5

Lourenco, M. V., Ferreira, S. T. & De Felice, F. G. Neuronal stress signaling and eIF2alpha phosphorylation as molecular links between Alzheimer's disease and diabetes. Progress in Neurobiology 129, 37-57 (2015). https://doi.org:https://dx.doi.org/10.1016/j.pneurobio.2015.03.003

Luciani, P. et al. Differentiating effects of the glucagon-like peptide-1 analogue exendin-4 in a human neuronal cell model. Cellular and Molecular Life Sciences 67, 3711-3723 (2010). https://doi.org:https://dx.doi.org/10.1007/s00018-010-0398-3

Ma, T. et al. Glucagon-like peptide-1 cleavage product GLP-1(9-36) amide rescues synaptic plasticity and memory deficits in Alzheimer's disease model mice. The Journal of Neuroscience 32, 13701-13708 (2012). https://doi.org:https://dx.doi.org/10.1523/JNEUROSCI.2107-12.2012

Martin, B. et al. Euglycemic agent-mediated hypothalamic transcriptomic manipulation in the N171-82Q model of huntington disease is related to their physiological efficacy. Journal of Biological Chemistry 287, 31766-31782 (2012). https://doi.org:https://dx.doi.org/10.1074/jbc.M112.387316

Maskery, M. et al. The dual GLP-1/GIP receptor agonist DA4-JC shows superior protective properties compared to the GLP-1 analogue liraglutide in the APP/PS1 mouse model of Alzheimer's disease. American Journal of Alzheimer's Disease and Other Dementias 35 (2020). https://doi.org:https://dx.doi.org/10.1177/1533317520953041

McClean, P. L. & Holscher, C. Lixisenatide, a drug developed to treat type 2 diabetes, shows neuroprotective effects in a mouse model of Alzheimer's disease. Neuropharmacology 86, 241-258 (2014). https://doi.org:https://dx.doi.org/10.1016/j.neuropharm.2014.07.015

McClean, P. L. & Holscher, C. Liraglutide can reverse memory impairment, synaptic loss and reduce plaque load in aged APP/PS1 mice, a model of Alzheimer's disease. Neuropharmacology 76, 57-67 (2014). https://doi.org:https://dx.doi.org/10.1016/j.neuropharm.2013.08.005

McClean, P. L., Jalewa, J. & Holscher, C. Prophylactic liraglutide treatment prevents amyloid plaque deposition, chronic inflammation and memory impairment in APP/PS1 mice. Behavioural Brain Research 293, 96-106 (2015). https://doi.org:https://dx.doi.org/10.1016/j.bbr.2015.07.024

McClean, P. L., Parthsarathy, V., Faivre, E. & Holscher, C. The diabetes drug liraglutide prevents degenerative processes in a mouse model of Alzheimer's disease. The Journal of Neuroscience 31, 6587-6594 (2011). https://doi.org:https://dx.doi.org/10.1523/JNEUROSCI.0529-11.2011

McGovern, S. F. J., Hunter, K. & Holscher, C. Effects of the glucagon-like polypeptide-1 analogue (Val8)GLP-1 on learning, progenitor cell proliferation and neurogenesis in the C57B/16 mouse brain. Brain Research 1473, 204-213 (2012). https://doi.org:https://dx.doi.org/10.1016/j.brainres.2012.07.029

Nistico, R., Pignatelli, M., Piccinin, S., Mercuri, N. B. & Collingridge, G. Targeting synaptic dysfunction in Alzheimer's disease therapy. Molecular Neurobiology 46, 572-587 (2012). https://doi.org:https://dx.doi.org/10.1007/s12035-012-8324-3

Nizari, S. et al. Glucagon-like peptide-1 (GLP-1) receptor activation dilates cerebral arterioles, increases cerebral blood flow, and mediates remote (pre)conditioning neuroprotection against ischaemic stroke. Basic Research in Cardiology 116, 32 (2021). https://doi.org:https://dx.doi.org/10.1007/s00395-021-00873-9

O'Neill, C. PI3-kinase/Akt/mTOR signaling: Impaired on/off switches in aging, cognitive decline and Alzheimer's disease. Experimental Gerontology 48, 647-653 (2013). https://doi.org:https://dx.doi.org/10.1016/j.exger.2013.02.025

Paladugu, L. et al. Liraglutide Has Anti-Inflammatory and Anti-Amyloid Properties in Streptozotocin-Induced and 5xFAD Mouse Models of Alzheimer's Disease. International journal of molecular sciences 22 (2021). https://doi.org:https://dx.doi.org/10.3390/ijms22020860

Palleria, C. et al. Liraglutide prevents cognitive decline in a rat model of streptozotocin-induced diabetes independently from its peripheral metabolic effects. Behavioural Brain Research 321, 157-169 (2017). https://doi.org:https://dx.doi.org/10.1016/j.bbr.2017.01.004

Panagaki, T. Neuroprotective effects of incretin mimetics upon chronic endoplasmic reticulum (ER) stress: Implications for novel therapeutic pathways and opportunities in Alzheimer's disease. Dissertation Abstracts International: Section B: The Sciences and Engineering 82, No-Specified (2021).

Panagaki, T., Gengler, S. & Holscher, C. The novel DA-CH3 dual incretin restores endoplasmic reticulum stress and autophagy impairments to attenuate Alzheimer-like pathology and cognitive decrements in the APPSWE/PS1DELTAE9 mouse model. Journal of Alzheimer's Disease 66, 195-218 (2018). https://doi.org:https://dx.doi.org/10.3233/JAD-180584

Park, J.-S. et al. Blocking microglial activation of reactive astrocytes is neuroprotective in models of Alzheimer's disease. Acta neuropathologica communications 9, 78 (2021). https://doi.org:https://dx.doi.org/10.1186/s40478-021-01180-z

Park, K. A. et al. Long-lasting exendin-4 fusion protein improves memory deficits in high-fat diet/streptozotocin-induced diabetic mice. Pharmaceutics 12, 159 (2020). https://doi.org:https://dx.doi.org/10.3390/pharmaceutics12020159

Parthsarathy, V. & Holscher, C. Chronic treatment with the GLP1 analogue liraglutide increases cell proliferation and differentiation into neurons in an AD mouse model. PLoS ONE 8 (2013). https://doi.org:https://dx.doi.org/10.1371/journal.pone.0058784

Peng, X. et al. Exendin-4 improves cognitive function of diabetic mice via increasing brain insulin synthesis. Current Alzheimer Research 18, 546-557 (2021). https://doi.org:https://dx.doi.org/10.2174/1567205018666210929150004

Perry, T., Haughey, N. J., Mattson, M. P., Egan, J. M. & Greig, N. H. Protection and reversal of excitotoxic neuronal damage by glucagon-like peptide-1 and exendin-4. Journal of Pharmacology and Experimental Therapeutics 302, 881-888 (2002). https://doi.org:https://dx.doi.org/10.1124/jpet.102.037481

Perry, T. et al. A novel neurotrophic property of glucagon-like peptide 1: A promoter of nerve growth factor-mediated differentiation in PC12 cells. Journal of Pharmacology and Experimental Therapeutics 300, 958-966 (2002). https://doi.org:https://dx.doi.org/10.1124/jpet.300.3.958

Perry, T. et al. Glucagon-like peptide-1 decreases endogenous amyloid-beta peptide (Abeta) levels and protects hippocampal neurons from death induced by Abeta and iron. Journal of Neuroscience Research 72, 603-612 (2003). https://doi.org:https://dx.doi.org/10.1002/jnr.10611

Perry, T. A. & Greig, N. H. The glucagon-like peptides: A new genre in therapeutic targets for intervention in Alzheimer's disease. Journal of Alzheimer's Disease 4, 487-496 (2002). https://doi.org:https://dx.doi.org/10.3233/JAD-2002-4605

Popelova, A. et al. Novel lipidized analog of prolactin-releasing peptide improves memory impairment and attenuates hyperphosphorylation of tau protein in a mouse model of tauopathy. Journal of Alzheimer's Disease 62, 1725-1736 (2018). https://doi.org:https://dx.doi.org/10.3233/JAD-171041

Qi, L. et al. Liraglutide reduces oxidative stress and improves energy metabolism in methylglyoxal-induced SH-SY5Y cells. Neurotoxicology 92, 166-179 (2022). https://doi.org:https://dx.doi.org/10.1016/j.neuro.2022.08.007

Qi, L. et al. Subcutaneous administration of liraglutide ameliorates learning and memory impairment by modulating tau hyperphosphorylation via the glycogen synthase kinase-3beta pathway in an amyloid beta protein induced alzheimer disease mouse model. European Journal of Pharmacology 783, 23-32 (2016). https://doi.org:https://dx.doi.org/10.1016/j.ejphar.2016.04.052

Qi, L. Q. et al. Subcutaneous liraglutide ameliorates methylglyoxal-induced alzheimer-like tau pathology and cognitive impairment by modulating tau hyperphosphorylation and glycogen synthase kinase-3beta. American Journal of Translational Research 9, 247-260 (2017).

Rebosio, C., Balbi, M., Passalacqua, M., Ricciarelli, R. & Fedele, E. Presynaptic GLP-1 receptors enhance the depolarization-evoked release of glutamate and GABA in the mouse cortex and hippocampus. BioFactors 44, 148-157 (2018). https://doi.org:https://dx.doi.org/10.1002/biof.1406

Robinson, A. et al. Combination of Insulin with a GLP1 Agonist Is Associated with Better Memory and Normal Expression of Insulin Receptor Pathway Genes in a Mouse Model of Alzheimer's Disease. Journal of molecular neuroscience : MN 67, 504-510 (2019). https://doi.org:https://dx.doi.org/10.1007/s12031-019-1257-9

Rocha-Ferreira, E. et al. Neuroprotective exendin-4 enhances hypothermia therapy in a model of hypoxic-ischaemic encephalopathy. Brain : a journal of neurology 141, 2925-2942 (2018). https://doi.org:https://dx.doi.org/10.1093/brain/awy220

Saad, M. A., Eltarzy, M. A., Abdel Salam, R. M. & Ahmed, M. A. E. Liraglutide mends cognitive impairment by averting Notch signaling pathway overexpression in a rat model of polycystic ovary syndrome. Life sciences 265, 118731 (2021). https://doi.org:https://dx.doi.org/10.1016/j.lfs.2020.118731

Safar, M. M., Shahin, N. N., Mohamed, A. F. & Abdelkader, N. F. Suppression of BACE1 and amyloidogenic/RAGE axis by sitagliptin ameliorates PTZ kindling-induced cognitive deficits in rats. Chemico-Biological Interactions 328, 109144 (2020). https://doi.org:https://dx.doi.org/10.1016/j.cbi.2020.109144

Salles, G. N. et al. Neuroprotective and restorative properties of the GLP-1/GIP dual agonist DA-JC1 compared with a GLP-1 single agonist in Alzheimer's disease. Neuropharmacology 162 (2020). https://doi.org:https://dx.doi.org/10.1016/j.neuropharm.2019.107813

Sannikova, E. P. et al. Specific Activity of Recombinant Modified Human Glucagon-Like Peptide 1. Applied Biochemistry and Microbiology 55, 722-732 (2019). https://doi.org:https://dx.doi.org/10.1134/S0003683819070068

Sedky, A. A. Improvement of cognitive function, glucose and lipid homeostasis and serum osteocalcin levels by liraglutide in diabetic rats. Fundamental & clinical pharmacology 35, 989-1003 (2021). https://doi.org:https://dx.doi.org/10.1111/fcp.12664

Sharma, M. K., Jalewa, J. & Holscher, C. Neuroprotective and anti-apoptotic effects of liraglutide on SH-SY5Y cells exposed to methylglyoxal stress. Journal of Neurochemistry 128, 459-471 (2014). https://doi.org:https://dx.doi.org/10.1111/jnc.12469

Shi, L., Zhang, Z., Li, L. & Holscher, C. A novel dual GLP-1/GIP receptor agonist alleviates cognitive decline by re-sensitizing insulin signaling in the Alzheimer icv. STZ rat model. Behavioural Brain Research 327, 65-74 (2017). https://doi.org:https://dx.doi.org/10.1016/j.bbr.2017.03.032

Siddiqui, N. et al. Linagliptin, a DPP-4 inhibitor, ameliorates Abeta (1-42) peptides induced neurodegeneration and brain insulin resistance (BIR) via insulin receptor substrate-1 (IRS-1) in rat model of Alzheimer's disease. Neuropharmacology 195 (2021). https://doi.org:https://dx.doi.org/10.1016/j.neuropharm.2021.108662

Song, X. et al. Exendin-4 alleviates beta-amyloid peptide toxicity via DAF-16 in a Caenorhabditis elegans model of Alzheimer's disease. Frontiers in Aging Neuroscience 14 (2022). https://doi.org:https://dx.doi.org/10.3389/fnagi.2022.955113

Spielman, L. J., Gibson, D. L. & Klegeris, A. Incretin hormones regulate microglia oxidative stress, survival and expression of trophic factors. European Journal of Cell Biology 96, 240-253 (2017). https://doi.org:https://dx.doi.org/10.1016/j.ejcb.2017.03.004

Spolcova, A. et al. Anorexigenic lipopeptides ameliorate central insulin signaling and attenuate tau phosphorylation in hippocampi of mice with monosodium glutamate-induced obesity. Journal of Alzheimer's Disease 45, 823-835 (2015).

Strnad, S. et al. Mass spectrometry imaging of free-floating brain sections detects pathological lipid distribution in a mouse model of Alzheimer's-like pathology. The Analyst 145, 4595-4605 (2020). https://doi.org:https://dx.doi.org/10.1039/d0an00592d

Su, Y. et al. A GLP-2 Analogue Protects SH-SY5Y and Neuro-2a Cells Against Mitochondrial Damage, Autophagy Impairments and Apoptosis in a Parkinson Model. Drug research 71, 43-50 (2021). https://doi.org:https://dx.doi.org/10.1055/a-1266-3263

Sun, H.-Z. et al. Exendin-4 increases the firing activity of hippocampal ca1 neurons through trpc4/5 channels. Neuroscience Research, No-Specified (2023). https://doi.org:https://dx.doi.org/10.1016/j.neures.2023.08.001

Tai, J., Liu, W., Li, Y., Li, L. & Holscher, C. Neuroprotective effects of a triple GLP-1/GIP/glucagon receptor agonist in the APP/PS1 transgenic mouse model of Alzheimer's disease. Brain Research 1678, 64-74 (2018). https://doi.org:https://dx.doi.org/10.1016/j.brainres.2017.10.012

Takach, O., Gill, T. B. & Silverman, M. A. Modulation of insulin signaling rescues BDNF transport defects independent of tau in amyloid-beta oligomer-treated hippocampal neurons. Neurobiology of Aging 36, 1378-1382 (2015). https://doi.org:https://dx.doi.org/10.1016/j.neurobiolaging.2014.11.018

Tan, Z. G., Gao, W. H., Cai, X. S., Wang, F. & Hui, H. X. Glucagon-like peptide 1 improves learning and memory abilities of rats with type 2 diabetes. Nan fang yi ke da xue xue bao = Journal of Southern Medical University 36, 1345-1351 (2016).

Trammell, T. S., Henderson, N. L., Madkour, H. S., Stanwood, G. D. & Graham, D. L. GLP-1R activation alters performance in cognitive tasks in a sex-dependent manner. Neurological Sciences 42, 2911-2919 (2021). https://doi.org:https://dx.doi.org/10.1007/s10072-020-04910-8

Tseng, Y. T., Lin, W. J., Chang, W. H. & Lo, Y. C. The novel protective effects of loganin against 1-methyl-4-phenylpyridinium-induced neurotoxicity: Enhancement of neurotrophic signaling, activation of IGF-1R/GLP-1R, and inhibition of RhoA/ROCK pathway. Phytotherapy Research 33, 690-701 (2019). https://doi.org:https://dx.doi.org/10.1002/ptr.6259

Velmurugan, K., Bouchard, R., Mahaffey, G. & Pugazhenthi, S. Neuroprotective actions of Glucagon-like peptide-1 in differentiated human neuroprogenitor cells. Journal of Neurochemistry 123, 919-931 (2012). https://doi.org:https://dx.doi.org/10.1111/jnc.12036

Vestlund, J., Bergquist, F., Licheri, V., Adermark, L. & Jerlhag, E. Activation of glucagon-like peptide-1 receptors and skilled reach foraging. Addiction Biology 26 (2021). https://doi.org:https://dx.doi.org/10.1111/adb.12953

Voss, U., Sand, E., Hellstrom, P. M. & Ekblad, E. Glucagon-like peptides 1 and 2 and vasoactive intestinal peptide are neuroprotective on cultured and mast cell co-cultured rat myenteric neurons. BMC Gastroenterology 12, 30 (2012). https://doi.org:https://dx.doi.org/10.1186/1471-230X-12-30

Walther, T. et al. Improved learning and memory in aged mice deficient in amyloid beta-degrading neutral endopeptidase. PloS one 4, e4590 (2009). https://doi.org:https://dx.doi.org/10.1371/journal.pone.0004590

Wang, G. et al. Exenatide exerts a neuroprotective effect against diabetic cognitive impairment in rats by inhibiting apoptosis: Role of the JNK/c-JUN signaling pathway. Molecular medicine reports 25 (2022). https://doi.org:https://dx.doi.org/10.3892/mmr.2022.12627

Wang, L. et al. Semaglutide attenuates seizure severity and ameliorates cognitive dysfunction by blocking the NLR family pyrin domain containing 3 inflammasome in pentylenetetrazole-kindled mice. International journal of molecular medicine 48 (2021). https://doi.org:https://dx.doi.org/10.3892/ijmm.2021.5052

Wang, L. et al. DA-JC1 improves learning and memory by antagonizing Abeta31-35-induced circadian rhythm disorder. Molecular Brain 12, 14 (2019). https://doi.org:https://dx.doi.org/10.1186/s13041-019-0432-9

Wang, M., Yoon, G., Song, J. & Jo, J. Exendin-4 improves long-term potentiation and neuronal dendritic growth in vivo and in vitro obesity condition. Scientific reports 11, 8326 (2021). https://doi.org:https://dx.doi.org/10.1038/s41598-021-87809-4

Wang, X. et al. Exendin-4 antagonizes Abeta1-42-induced suppression of long-term potentiation by regulating intracellular calcium homeostasis in rat hippocampal neurons. Brain Research 1627, 101-108 (2015). https://doi.org:https://dx.doi.org/10.1016/j.brainres.2015.09.015

Wang, X.-H. et al. Val8-GLP-1 remodels synaptic activity and intracellular calcium homeostasis impaired by amyloid beta peptide in rats. Journal of Neuroscience Research 91, 568-577 (2013). https://doi.org:https://dx.doi.org/10.1002/jnr.23181

Wang, Y. et al. GLP-1 receptor agonists downregulate aberrant GnT-III expression in Alzheimer's disease models through the Akt/GSK-3beta/beta-catenin signaling. Neuropharmacology 131, 190-199 (2018). https://doi.org:https://dx.doi.org/10.1016/j.neuropharm.2017.11.048

Wang, Z. et al. Mollugin activates GLP-1R to improve cognitive dysfunction in type 2 diabetic mice. Life Sciences 331, 122026 (2023). https://doi.org:https://dx.doi.org/10.1016/j.lfs.2023.122026

Wang, Z.-J. et al. A dual GLP-1 and Gcg receptor agonist rescues spatial memory and synaptic plasticity in APP/PS1 transgenic mice. Hormones and Behavior 118 (2020). https://doi.org:https://dx.doi.org/10.1016/j.yhbeh.2019.104640

Wang, Z.-J. et al. Semaglutide ameliorates cognition and glucose metabolism dysfunction in the 3xTg mouse model of Alzheimer's disease via the GLP-1R/SIRT1/GLUT4 pathway. Neuropharmacology 240, 1-15 (2023). https://doi.org:https://dx.doi.org/10.1016/j.neuropharm.2023.109716

Watson, K. T. et al. Neural correlates of liraglutide effects in persons at risk for Alzheimer's disease. Behavioural Brain Research 356, 271-278 (2019). https://doi.org:https://dx.doi.org/10.1016/j.bbr.2018.08.006

Wicinski, M. et al. Liraglutide and its Neuroprotective Properties-Focus on Possible Biochemical Mechanisms in Alzheimer's Disease and Cerebral Ischemic Events. International journal of molecular sciences 20 (2019). https://doi.org:https://dx.doi.org/10.3390/ijms20051050

Xie, Y. et al. GLP-1 improves the neuronal supportive ability of astrocytes in Alzheimer's disease by regulating mitochondrial dysfunction via the cAMP/PKA pathway. Biochemical pharmacology 188, 114578 (2021). https://doi.org:https://dx.doi.org/10.1016/j.bcp.2021.114578

Xie, Y.-C. et al. Glucagon-like peptide-2 receptor is involved in spatial cognitive dysfunction in rats after chronic cerebral hypoperfusion. Journal of Alzheimer's Disease 66, 1559-1576 (2018). https://doi.org:https://dx.doi.org/10.3233/JAD-180782

Xiong, H. et al. The neuroprotection of liraglutide on Alzheimer-like learning and memory impairment by modulating the hyperphosphorylation of tau and neurofilament proteins and insulin signaling pathways in mice. Journal of Alzheimer's Disease 37, 623-635 (2013). https://doi.org:https://dx.doi.org/10.3233/JAD-130584

Yan, W. et al. The neuroprotection of liraglutide on diabetic cognitive deficits is associated with improved hippocampal synapses and inhibited neuronal apoptosis. Life sciences 231, 116566 (2019). https://doi.org:https://dx.doi.org/10.1016/j.lfs.2019.116566

Yang, Y. et al. Subcutaneous administration of liraglutide ameliorates Alzheimer-associated tau hyperphosphorylation in rats with type 2 diabetes. Journal of Alzheimer's Disease 37, 637-648 (2013). https://doi.org:https://dx.doi.org/10.3233/JAD-130491

Yin, F., Zhang, Y., Guo, L., Kong, S. & Liu, J. Geniposide regulates insulin-degrading enzyme expression to inhibit the cytotoxicity of Abeta1-42 in cortical neurons. CNS and Neurological Disorders - Drug Targets 11, 1045-1051 (2012). https://doi.org:https://dx.doi.org/10.2174/1871527311211080015

Yoshino, Y., Ishisaka, M., Tsujii, S., Shimazawa, M. & Hara, H. Glucagon-like peptide-1 protects the murine hippocampus against stressors via Akt and ERK1/2 signaling. Biochemical and Biophysical Research Communications 458, 274-279 (2015). https://doi.org:https://dx.doi.org/10.1016/j.bbrc.2015.01.098

Yu, C. J. et al. The role of GLP-1/GIP receptor agonists in Alzheimer's disease. Advances in clinical and experimental medicine : official organ Wroclaw Medical University 29, 661-668 (2020). https://doi.org:https://dx.doi.org/10.17219/acem/121007

Yuan, Z. et al. A novel GLP-1/GIP dual agonist is more effective than liraglutide in reducing inflammation and enhancing GDNF release in the MPTP mouse model of Parkinson's disease. European Journal of Pharmacology 812, 82-90 (2017). https://doi.org:https://dx.doi.org/10.1016/j.ejphar.2017.06.029

Zanotto, C. et al. Glutamatergic Alterations in STZ-Induced Diabetic Rats Are Reversed by Exendin-4. Molecular neurobiology 56, 3538-3551 (2019). https://doi.org:https://dx.doi.org/10.1007/s12035-018-1320-5

Zhang, H. et al. Liraglutide improved the cognitive function of diabetic mice via the receptor of advanced glycation end products down-regulation. Aging 13, 525-536 (2020). https://doi.org:https://dx.doi.org/10.18632/aging.202162

Zhang, H. et al. Pro-GLP-1, a Pro-drug of GLP-1, is neuroprotective in cerebral ischemia. European Journal of Pharmaceutical Sciences 70, 82-91 (2015). https://doi.org:https://dx.doi.org/10.1016/j.ejps.2015.01.010

Zhang, H. et al. Glucagon-like peptide-1 attenuated carboxymethyl lysine induced neuronal apoptosis via peroxisome proliferation activated receptor-gamma. Aging 13, 19013-19027 (2021). https://doi.org:https://dx.doi.org/10.18632/aging.203351

Zhang, J. et al. Liraglutide Nano-Preparation on Perioperative Neurocognitive Dysfunction in Aged Mice. Cellular and molecular biology (Noisy-le-Grand, France) 68, 356-364 (2022). https://doi.org:https://dx.doi.org/10.14715/cmb/2022.68.3.39

Zhang, L.-Q. et al. GLP-1R activation ameliorated novel-object recognition memory dysfunction via regulating hippocampal AMPK/NF-kappaB pathway in neuropathic pain mice. Neurobiology of Learning and Memory 182 (2021). https://doi.org:https://dx.doi.org/10.1016/j.nlm.2021.107463

Zhang, M. et al. Glucagon-like peptide-1 analogs mitigate neuroinflammation in Alzheimer's disease by suppressing NLRP2 activation in astrocytes. Molecular and cellular endocrinology 542, 111529 (2022). https://doi.org:https://dx.doi.org/10.1016/j.mce.2021.111529

Zhang, M. et al. Liraglutide ameliorates diabetes-associated cognitive dysfunction via rescuing autophagic flux. Journal of pharmacological sciences 147, 234-244 (2021). https://doi.org:https://dx.doi.org/10.1016/j.jphs.2021.07.004

Zhang, Q., Jiang, Y., Zhang, Y., Zhang, J. & Hu, Y. GLP-1 Analog Alleviated Cognitive Dysfunction in Aged Rats Anaesthetized with Sevoflurane. BioMed research international 2022, 9339824 (2022). https://doi.org:https://dx.doi.org/10.1155/2022/9339824

Zhang, S.-X. et al. GLP-1 analogue CJC-1131 prevents amyloid beta protein-induced impirments of spatial memory and synaptic plasticity in rats. Behavioural Brain Research 326, 237-243 (2017). https://doi.org:https://dx.doi.org/10.1016/j.bbr.2017.03.018

Zhang, Y. et al. Liraglutide Ameliorates Hyperhomocysteinemia-Induced Alzheimer-Like Pathology and Memory Deficits in Rats via Multi-molecular Targeting. Neuroscience bulletin 35, 724-734 (2019). https://doi.org:https://dx.doi.org/10.1007/s12264-018-00336-7

Zhang, Z. et al. A Dual GLP-1/GIP Receptor Agonist Is More Effective than Liraglutide in the A53T Mouse Model of Parkinson's Disease. Parkinson's disease 2023, 7427136 (2023). https://doi.org:https://dx.doi.org/10.1155/2023/7427136

Zhao, L. et al. Pharmacologically reversible zonation-dependent endothelial cell transcriptomic changes with neurodegenerative disease associations in the aged brain. Nature communications 11, 4413 (2020). https://doi.org:https://dx.doi.org/10.1038/s41467-020-18249-3

Zheng, C. et al. The protective effects of liraglutide on AD-like neurodegeneration induced by oxidative stress in human neuroblastoma SH-SY5Y cells. Chemico-biological interactions 310, 108688 (2019). https://doi.org:https://dx.doi.org/10.1016/j.cbi.2019.06.001

Zheng, M. & Wang, P. Role of insulin receptor substance-1 modulating PI3K/Akt insulin signaling pathway in Alzheimer's disease. 3 Biotech 11, 179 (2021). https://doi.org:https://dx.doi.org/10.1007/s13205-021-02738-3

Zhou, M. et al. Dulaglutide ameliorates STZ induced AD-like impairment of learning and memory ability by modulating hyperphosphorylation of tau and NFs through GSK3beta. Biochemical and biophysical research communications 511, 154-160 (2019). https://doi.org:https://dx.doi.org/10.1016/j.bbrc.2019.01.103

S5. Pre-clinical/mechanistic studies on cognitive disorders, substance use disorders, psychotic disorders, mood and anxiety disorders, and eating disorders

Supplementary Table S5. Summary of included pre-clinical/mechanistic studies regarding the effects of GLP1-RAs on mental health outcomes.

Study ID	Sample	Model	Intervention	Main Finding(s)	
Cognitive disorders					
Erbil 2019	Review of pre-clinical studies		Any GLP1-RA	GLP-1RAs may reverse neurovascular complications of diabetes and neuropathological changes related to AD, PD or vascular occlusion. Neuroprotective mechanisms include: enhancing the viability of neurons and restoring neurite outgrowth by increased neurotrophic factors, increasing subventricular zone progenitor cells, decreasing apoptosis, decreasing the level of pro-inflammatory factors, and strengthening the blood-brain barrier.	NA
Kong 2023	Review of pre-clinical studies		Any GLP1-RA	GLP-1 RAs improve the learning and memory abilities of AD rodents and reduce Aβ deposition and phosphorylated tau levels in the brains of AD rodents.	NA
Nowell 2023	Review of pre-clinical studies		Any GLP1-RA	GLP-1 RAs reduce neuroinflammation, reduce tau phosphorylation, reduce Aβ deposition, increase synaptic function, and improve memory formation.	NA
Reich 2022	Review of pre-clinical studies		Any GLP1-RA	Neuroprotective pathways induced following GLP-1R activation in neurons, microglia and astrocytes include: synaptic protection, Aβ pathology amelioration, suppression of Ca^{2+} deregulation and ER stress, potent anti-inflammatory effects, the blockage of oxidative stress, mitochondrial dysfunction and apoptosis pathways, enhancements in the neuronal insulin sensitivity and energy metabolism, functional improvements in autophagy and mitophagy, elevated BDNF and GDNF synthesis, as well as neurogenesis.	NA
Substance use disorders					
Alcohol					
Abtahi 2018	Animal	Rats	Exendin-4	Exendin-4 administration in the NAc decreases alcohol consumption in alcohol-habituated rats.	+
Allingbjerg 2023	Animal	Mice	Exendin-4	Exendin-4 infusion into the NAc, ventral hippocampus and lateral septum reduced alcohol self-administration.	+
Aranas 2023a	Animal	Rats	Semaglutide	Semaglutide reduces alcohol intake and prevents relapse-like drinking via dopaminergic mechanisms in the NAc.	+
Aranas 2023b	Animal	Rats	Semaglutide + Bupropion / Varenicline	No additive effects on alcohol intake reduction by adding antismoking agents (bupropion or varenicline) to semaglutide.	=
Bornebusch 2019	Animal	Mice	Exendin-4	Exendin-4 dose-dependently decreases oral alcohol self-administration.	+
Chuong 2023	Animal	Mice and rats	Semaglutide	Semaglutide reduces binge-like alcohol drinking in mouse and rat models of alcohol use disorder models.	+
Colvin 2020	Animal	Rats	Exendin-4	VTA, NAc and lateral hypothalamus are involved in the inhibition of alcohol intake by Exendin-4.	+
Colvin 2022	Animal	Rats	Exendin-4	VTA exendin-4 inhibits alcohol intake and reverses the stimulatory effect of cocaine and d-amphetamine on alcohol consumption.	+

Davis 2012	Animal	Rats	Exendin-4	GLP-1 agonism attenuates alcohol intake in rats selectively bred to consume alcohol. Roux-en-Y gastric bypass surgery in rats decreases alcohol intake by increasing GLP-1 levels.	+
Diaz-Megido 2023	Animal	Mice	Exendin-4	Suppression of alcohol reinforcement and reinstatement by Exendin-4 in male mice.	+
Dixon 2020	Animal	Rats	Exendin-4	VTA administration of exendin-4 inhibits alcohol self-administration in male rats.	+
Farokhnia 2022	Human	Post-mortem brain samples of patients with severe AUD	-	Increased hippocampal expression of GLP-1R gene in post-mortem tissue from individuals with AUD versus controls.	+
Egecioglu 2013	Animal	Mice	Exendin-4	Exendin-4 reduces alcohol-induced locomotor stimulation, DA release in the NAc shell, and attenuates the reinforcing properties of alcohol.	+
Marty 2020	Animal	Rats	Liraglutide<br>Semaglutide	Transient reduction in voluntary alcohol intake by liraglutide or semaglutide.	+
Sharma 2015a	Animal	Rats	Liraglutide	Liraglutide inhibits withdrawal-induced anxiety and prevents tolerance to the anxiolytic effect of alcohol.	+
Shirazi 2013	Animal	Rats	Exendin-4	Peripheral or VTA injection of exendin-4 reduces alcohol intake.	+
Sorensen 2016	Animal	Mice	Exendin-4	Pre-treatment with exendin-4 attenuates voluntary alcohol self-administration.	+
Suchankova 2015	Animal	Mice	AC3174 ([Leu14]-exendin-4)	GLP-1R agonism significantly reduces alcohol consumption in a mouse model of alcohol dependence.	+
Thomsen 2017	Animal	Mice	Exendin-4	Treatment with exendin-4 during alcohol deprivation decreases relapse-like drinking in socially housed mice.	+
Thomsen 2019	Animal	African vervet monkeys	Exenatide<br>Liraglutide	Liraglutide and, to a lesser extent, exenatide reduce voluntary alcohol consumption in non-human primates.	+
Vallof 2016	Animal	Rats	Liraglutide	Liraglutide suppresses alcohol-induced effects on the mesolimbic DA system and attenuates the reinforcing properties of alcohol.	+
Vallof 2019a	Animal	Mice and rats	Exendin-4	Exendin-4 prevents alcohol-induced locomotion, suppresses memory of alcohol reward or reduces alcohol intake depending on the brain region it is infused into.	+
Vallof 2019b	Animal	Mice and rats	Exendin-4	Exendin-4 infusion into the NST inhibits alcohol-induced locomotion, accumbal DA release, CPP-dependent alcohol memory in mice, and alcohol intake in rats.	+
Vallof 2020	Animal	Rats	Dulaglutide	Long-term dulaglutide reduces alcohol intake and preference, which was sustained after discontinuation in males, but not females.	+
Cocaine/Amphetamines					
Bouhlal 2017	Human	8 experienced cocaine users	IV cocaine	IV cocaine decreases plasma GLP-1 concentration. Endogenous GLP-1 is associated with subjective responses to cocaine.	+
Egecioglu 2013b	Animal	Mice	Exendin-4	Exendin-4 reduces amphetamine- and cocaine-induced locomotor stimulation, accumbal DA release and CPP.	+
Erreger 2012	Animal	Rats	Exendin-4	Single acute dose of exendin-4 decreases amphetamine-induced locomotor activity.	+
Fortin 2017	Animal	Rats	Exendin-4	Central exendin-4 suppresses cocaine-induced phasic DA signalling in the NAc core which may decrease the reinforcing properties of cocaine.	+
Graham 2013	Animal	Mice	Exendin-4	Exendin-4 attenuates cocaine-induced CPP (but even at the highest doses did not eliminate CPP).	+

Harasta 2015	Animal	Mice	AAV-mediated Glp-1r gene delivery to the dorsal lateral septum	Septal Glp-1r gene expression in Glp-1r−/− animals reduces cocaine-induced locomotion and CPP to wild-type levels.	+
Hernandez 2018	Animal	Rats	Exendin-4	Exendin-4 reduces cocaine seeking through effects on the VTA.	+
Hernandez 2019	Animal	Rats	Exendin-4	Increased activation of GLP-1R in the NA during cocaine abstinence is sufficient to reduce cocaine-seeking behaviour.	+
Hernandez 2021	Animal	Rats	Exendin-4	Exendin-4 attenuates cocaine seeking via GABAergic GLP-1R-expressing circuits in the midbrain.	+
Reddy 2016	In Vitro	Rat lateral septum slices	Exendin-4	Exendin-4 abolishes cocaine-induced elevation of DA in lateral septum slices by decreasing arachidonic acid levels.	+
Schmidt 2016	Animal	Rats	Exendin-4	VTA administration of exendin-4 reduces cocaine self-administration.	+
Sirohi 2016	Animal	Mice	Exendin-4	Amphetamine-induced CPP was completely blocked in FLOX, but not in GLP-1R KD mice by exendin-4.	+
Sorensen 2015	Animal	Mice	Exendin-4	GLP-1R stimulation reduces acute and chronic cocaine self-administration and attenuates cocaine-induced hyperlocomotion.	+
Zhu 2021a	Animal	Mice	Exendin-4	Exendin-4 facilitates the extinction of cocaine-induced CPP.	+
Zhu 2021b	Animal	Mice	Exendin-4	Exendin-4 ameliorates cocaine-induced behaviours through inhibition of TLR4, TNF-α, and IL-1β.	+
Zhu 2021c	Animal	Mice	Exendin-4	Exendin-4 attenuates cocaine- and stress-primed reinstatement of cocaine-induced CPP.	+
Opiates					
Bornebusch 2019	Animal	Mice	Exendin-4	Exendin-4 did not attenuate morphine-induced CPP, withdrawal or hyperlocomotion, and did not decrease remifentanil self-administration.	=
Douton 2022a	Animal	Rats with heroin self-administration experience	Liraglutide	Acute dose of liraglutide prevents cue-, stress-, and drug-induced heroin-seeking in rats.	+
Douton 2022b	Animal	Rats	Liraglutide	Daily treatment with liraglutide reduces heroin self-administration and drug-induced reinstatement of heroin-seeking behaviour in rats.	+
Douton 2021	Animal	Rats with reward devaluation model	Exendin-4	Exendin-4 decreases cue- and drug-induced heroin seeking.	+
Evans 2022	Animal	Rats with history of high drug-taking	Liraglutide	Liraglutide titration decreases cue- and drug-induced heroin seeking	+
Urbanik 2022	Animal	Rats	Liraglutide	Acute dose of liraglutide attenuates cue-induced fentanyl-seeking and drug-induced reinstatement of fentanyl-seeking with the same efficacy as buprenorphine.	+
Zhang 2020	Animal	Rats	Exendin-4	Administration of exendin-4 systematically or into the NAc shell reduces oxycodone self-administration and the reinstatement of oxycodone-seeking behaviour.	+
Zhang 2021	Animal	Rats fentanyl-experienced	GEP44 (dual agonist of GLP-1Rs and neuropeptide Y2 receptors)	GEP44 attenuates opioid taking and seeking at a dose that does not suppress food intake or produce adverse malaise-like effects.	+
Nicotine					
Egecioglu 2013c	Animal	Mice	Exendin-4	Exendin-4 attenuates the nicotine-induced effects on the mesolimbic DA system (locomotor stimulation, accumbal DA release, CPP, expression of locomotor sensitisation).	+

Falk 2023	Animal	Mice obese	Liraglutide	Co-administration of nicotine and liraglutide reduces body weight and increases DA neuron excitability in the VTA. Liraglutide inhibits nicotine-induced DA release in the Nac.	+
Herman 2023	Animal	Rats	Liraglutide	Liraglutide attenuates nicotine self-administration and reinstatement, as well as withdrawal-induced hyperphagia.	+
Tuesta 2017	Animal	Mice	Optogenetic stimulation of GLP-1R	Optogenetic activation of GLP-1Rs in habenular circuits abolished nicotine reward and decreased nicotine intake.	+
Psychotic disorders					
Cardiometabolic side effects of antipsychotics					
Babic 2018	Animal	Rats treated with olanzapine or clozapine	Liraglutide	Liraglutide co-treatment improves aspects of cognition (recognition and working memory), prevents obesity side-effects of olanzapine, and hyperglycaemia caused by clozapine.	+
Babic 2021	Animal	Rats treated with olanzapine or clozapine	Liraglutide	Chronic APD and liraglutide co-treatment did not alter neural and peripheral markers of metabolic function. Acute liraglutide co-treatment did not prevent clozapine-induced hyperglycaemia.	=
Klemettila 2021	Human	190 patients with schizophrenia on clozapine treatment	-	Serum GLP-1 levels are associated with metabolic risk markers (BMI, leptin, insulin) among men with schizophrenia on clozapine treatment.	NA
Li 2021b	Animal	Rats treated with brexpiprazole	Liraglutide	Liraglutide ameliorates brexpiprazole-caused glycolipid metabolic abnormalities.	+
Lykkegaard 2008	Animal	Rats treated with olanzapine	Liraglutide	Olanzapine-induced weight gain and metabolic changes were reversed by liraglutide treatment.	+
Mansur 2019	Human	Post-mortem brain tissue of patients with mood and psychotic disorders	-	Significant differences in dlPFC and hippocampal GLP-1R gene expression between healthy controls and MD post-mortem subjects, moderated by BMI.	NA
Medak 2020	Animal	Mice treated with olanzapine	Liraglutide, Exendin-4	GLP1-R agonism protects against acute olanzapine-induced hyperglycaemia.	+
Sharma 2015b	Animal	Rats treated with olanzapine	Liraglutide	Liraglutide partially reversed metabolic abnormalities and depression-like behaviour associated with long-term olanzapine treatment.	+
Smith 2009	Animal	Obese rats treated with olanzapine or quetiapine	Exendin-4	Exendin-4 normalised both glucagon levels and glucose metabolism in clozapine or quetiapine-treated obese rats.	+
Smith 2014	Animal	Mice treated with clozapine	Boc5 (non-peptidic GLP1-RA)	Boc5 can overcome the inhibitory effects of clozapine on glucose metabolism.	+
Effects on psychosis					
Bocchio-Chiavetto 2018	Human	260 patients with FEP	-	Significantly lower levels of serum GLP-1 in FEP patients compared to healthy controls.	NA
Dixit 2013	Animal	Mouse model of psychosis induced by apomorphine	Liraglutide	Antipsychotic-like effect of liraglutide significantly attenuated apomorphine-induced cage climbing.	+
Kutlu 2023	Animal	Mouse schizophrenia model induced by MK-801	Liraglutide	Liraglutide prevents MK-801-induced schizophrenia-like behaviours and BDNF, CREB, p-CREB, Trk-B expressions in the hippocampus and prefrontal cortex.	+
Ramsey 2014	Human	Patients on antipsychotics from the CATIE trial	Various APDs	GLP-1R haplotypes significantly correlate with reduction in response to multiple antipsychotics as measured by dPANSS.	NA
Sedky 2021	Animal	Diabetic and non-diabetic rats with ketamine-induced psychotic-like behaviours	Liraglutide	Beneficial effects of liraglutide on ketamine-induced hyperlocomotion and cognitive dysfunction associated with reduction in TNF-α and oxidative stress, in both diabetic and non-diabetic rats.	+

Mood and anxiety disorders					
GLP -1RA effects on mood symptoms					
Anderberg 2016	Animal	Rats	Exendin-4	Chronic central administration of Exendin-4 does not alter anxiety-like behaviour but significantly reduces depression-like behaviour in the forced swim test.	+ (depression) = (anxiety)
Aygun 2021	Animal	WAG/Rij rat - genetic model of absence epilepsy and depression-like comorbidity	Exendin-4	Exendin-4 increases incidence of absence-like seizures, as well as anxiety- and depression-like behaviours.	-
Cicekli 2022	Animal	Ouabain-induced bipolar disease model in rats	Liraglutide + Sodium valproate	Ouabain-induced mania and depressive-like behaviours are ameliorated by liraglutide, which exerts antioxidant action, possibly improving GSK-3β phosphorylation.	+
Chaves Filho 2020	Animal	Mice with D-amphetamine-induced mania	Liraglutide +/- Lithium	Liraglutide reverses mania-like alterations and memory deficits induced by D-amphetamine and augments lithium effects in mice.	+
Darwish 2023	Animal	Social defeat stress model in C57BL/6 male mice	Dulaglutide	Dulaglutide reduces depressive behaviours by activating the GLP-1R/cAMP/PKA pathway. Social interaction ratio, sucrose consumption, and exploration time in open arms (in the EPM test) significantly increased after the administration of Dulaglutide.	+
DeSouza 2019	Animal	Mice subjected to PTZ-induced kindling (model for epileptogenesis)	Liraglutide +/- Levetiracetam	Liraglutide prevents the depressive-like behaviour induced by PTZ kindling or by PTZ + levetiracetam.	+
Krass 2015	Animal	Mice / Flinders sensitive line (FSL) rats	Exenatide / Liraglutide	Exenatide or liraglutide do not induce anxiolytic or antidepressant-like effects in mice in the FST. Chronic liraglutide treatment does not affect depression-related behaviour in FSL rats.	=
López-Ferreras 2020	Animal	Male and female rats	Exendin-4	Microinjecting exendin-4 into the SuM has anxiogenic effects which persist after exposure to high-fat diet. Knocking down GLP-1R expression in the SuM is anxiolytic in females only.	-
Ren 2021	Animal	CUMS mouse model of depression	Lixisenatide	Intranasal lixisenatide improves depressive and anxiety symptoms, possibly by increasing phosphorylation of CREB protein in hippocampal tissue.	+
Saglam 2022	Animal	Rats with ovariectomy-induced behavioural despair and anxiety-like behaviours	Liraglutide	Liraglutide reduces behavioural despair and anxiety-like behaviour, possibly through preservation of BDNF/Nrf2 levels and decrease in oxidative stress in the hippocampus.	+
Seo 2023	Animal	CUMS mouse model of depression	Liraglutide	Liraglutide exerts antidepressant effects and could improve cognitive function.	
Turan 2021	Animal	Mice subjected to REM sleep deprivation	Exenatide	Treatment with exenatide did not affect depression-like behaviour in the FST.	=
Verovnik 2023	Human	18 women with obesity or PCOS	Semaglutide	No significant change compared to placebo in fMRI resting state functional connectivity in regions of interest related to suicidal ideation and MD behaviours at 16 weeks	=
Weina 2018	Animal	CORT mouse model of depression	Liraglutide	Liraglutide administration attenuates depressive- and anxiety-like behaviours in behavioural tasks.	+
Yang 2022	Animal	Mice diabetic	Exendin-4	Exendin-4 exhibits antidepressant effects in depression associated with diabetes i, which may be mediated by inhibiting microglial pyroptosis via promoting mitophagy.	+
Other related agonists and their effects on depression models / other comorbid conditions					
Sun 2021	Animal	Diabetes-associated depression mouse model	Geniposide	Geniposide treatment improves cognitive dysfunctions and depressive/anxiety symptoms.	+

Hu 2021	Animal	Mice subjected to HFD which induces T2DM and depressive-like symptoms	Puerarin	Puerarin ameliorates depressive symptoms, possibly through activating the GLP-1R/Wnt/mTOR signalling pathway and improving hippocampal neuroplasticity.	+
Iwai 2009	Animal	Mice	GLP-2	GLP-2 exerts antidepressant-like effects in the FST.	+
Iwai 2013	Animal	ACTH-treated mice as a model of TCA-resistant depression	GLP-2	GLP-2 induces antidepressant-like effects in the FST, decreases serum corticosterone levels after the FST, and increases 5-HT levels in the frontal cortex.	+
Liu 2023	Animal	Diabetic mice	Puerarin	Taken together, puerarin exerts anti-depressant-like effects on HFD diabetic mice by improving hippocampal neuroplasticity via GLP-1R/BDNF/TrkB signalling.	+
Zhao 2018	Animal	RRS-induced depression model in mice	Geniposide	Geniposide ameliorates depression-like behaviours, such as decreased sucrose preference, reduced locomotor activity, and extended immobility time on the FST.	+
Eating disorders					
Cao 2014	Animal	Ovarectomised female mice	GLP-1	GLP-1 alone and GLP-1-estrogen suppress binge eating in ovariectomised female mice.	+
Mukherjee 2020	Animal	Rats	-	Binge-like palatable food intake reduces expression of GLP-1R in the NST of rats.	NA
Pierce-Messick 2020	Animal	Rats	Exendin-4	Exendin-4 reduces binge-like feeding induced by NAc □-opioid receptor stimulation.	+
Yamaguchi 2017	Animal	Mouse model of binge eating	GLP-1	Systemic GLP-1 reduces binge-like sucrose overconsumption.	+

Legend: + : positive effect; = : no effect; - : negative effect. Study ID reports the first author and year only.
Aβ: Amyloid Beta; ACTH: Adrenocorticotropic hormone; AD: Alzheimer's Disease; APD: Anti-Psychotic Drugs; AUD: Alcohol Use Disorder; BDNF: Brain-Derived Neurotrophic Factor; cAMP: Cyclic Adenosine Monophosphate; CORT: Chronic Corticosterone; CPP: Conditioned Place Preference; CREB: cAMP Response Element-Binding Protein; CUMS: Chronic unpredictable mild stress; DA: Dopamine; dlPFC: dorsolateral prefrontal cortex; EPM: Elevated Plus Maze; ER: Endoplasmic Reticulum; FEP: First Episode Psychosis; FST: Forced Swim Test; GDNF: Glial cell line-Derived Neurotrophic Factor; GLP-1RA: Glucagon-Like Peptide-1 Receptor Agonist; GSK-3β: Glycogen synthase kinase-3 beta; HFD: High-Fat Diet; IL-1β: Interleukin-1 beta; KD: Knock Down; MD: Major Depression; NA: Not Applicable; NAc: Nucleus Accumbens; NST: nucleus of the solitary tract; dPANSS: Positive and Negative Syndrome Scale (Total); PKA: Protein Kinase A; PTZ: Pentylenetetrazole; RRS: Repeated Restraint Stress; SuM: supramammillary nucleus; TCA: Tricyclic Antidepressant; T2DM: Type 2 Diabetes Mellitus; TLR4: Toll-Like Receptor 4; TrkB: Tyrosine Receptor Kinase B; TNF-α: Tumour Necrosis Factor alpha; VTA: Ventral Tegmental Area.
